# Structural and Functional Characterization of Anti-A33 Antibodies Reveal a Potent Cross-Species Orthopoxviruses Neutralizer

**DOI:** 10.1371/journal.ppat.1005148

**Published:** 2015-09-01

**Authors:** Michael H. Matho, Andrew Schlossman, Xiangzhi Meng, Mohammed Rafii-El-Idrissi Benhnia, Thomas Kaever, Mark Buller, Konstantin Doronin, Scott Parker, Bjoern Peters, Shane Crotty, Yan Xiang, Dirk M. Zajonc

**Affiliations:** 1 Division of Cell Biology, La Jolla Institute for Allergy and Immunology, La Jolla, California, United States of America; 2 Department of Microbiology and Immunology, University of Texas Health Science Center, San Antonio, Texas, United States of America; 3 Department of Medical Biochemistry, Molecular Biology, and Immunology, School of Medicine, University of Seville; and Laboratory of Immunovirology, Unit 211, Biomedicine Institute of Seville (IBIS), Seville, Spain; 4 Division of Vaccine Discovery, La Jolla Institute for Allergy and Immunology, La Jolla, California, United States of America; 5 Saint Louis University School of Medicine, St. Louis, Missouri, United States of America; University of Alberta, CANADA

## Abstract

Vaccinia virus A33 is an extracellular enveloped virus (EEV)-specific type II membrane glycoprotein that is essential for efficient EEV formation and long-range viral spread within the host. A33 is a target for neutralizing antibody responses against EEV. In this study, we produced seven murine anti-A33 monoclonal antibodies (MAbs) by immunizing mice with live VACV, followed by boosting with the soluble A33 homodimeric ectodomain. Five A33 specific MAbs were capable of neutralizing EEV in the presence of complement. All MAbs bind to conformational epitopes on A33 but not to linear peptides. To identify the epitopes, we have adetermined the crystal structures of three representative neutralizing MAbs in complex with A33. We have further determined the binding kinetics for each of the three antibodies to wild-type A33, as well as to engineered A33 that contained single alanine substitutions within the epitopes of the three crystallized antibodies. While the Fab of both MAbs A2C7 and A20G2 binds to a single A33 subunit, the Fab from MAb A27D7 binds to both A33 subunits simultaneously. A27D7 binding is resistant to single alanine substitutions within the A33 epitope. A27D7 also demonstrated high-affinity binding with recombinant A33 protein that mimics other orthopoxvirus strains in the A27D7 epitope, such as ectromelia, monkeypox, and cowpox virus, suggesting that A27D7 is a potent cross-neutralizer. Finally, we confirmed that A27D7 protects mice against a lethal challenge with ectromelia virus.

## Author Summary

Before the eradication of smallpox (variola virus) from nature, hundreds of million of people succumbed to the infection. The discovery of vaccinia virus (VACV), the active ingredient of the smallpox vaccine, ultimately led to the eradiation of smallpox from the human population. Vaccination with VACV leads to a strong antibody response that protects against variola virus. As the protective antibodies recognize viral proteins that are highly similar in sequence between the different orthopox strains, such as A33 used in this study, several antibodies have the capacity to neutralize a larger breath of orthopx viruses. In this study we have identified an anti-A33 antibody from a larger panel that exhibits a unique binding mode to A33. This antibody, A27D7, is also resistant to single amino acid changes throughout the protein and binds to engineered A33 variants that mimic ectromelia and orthopox A33 in the antibody-binding site. As the antibody further protects against ectromelia infection of mice, this antibody appears to be a potent orthopox cross-species protective antibody with therapeutic potential.

## Introduction

Inoculation with vaccinia virus (VACV) protected against smallpox, a deadly disease caused by the related orthopoxvirus, variola (VARV) [1]. Its success pertains to its high infectivity and thermal stability, and the strong innate and B-cell immune responses that it triggers [2, 3]. With the eradication of circulating variola virus from the human population, large-scale vaccination efforts against smallpox were ended [4, 5]. As a result, the general population is no longer protected against orthopoxviruses. This lack of immunity is a concern due to the zoonotic risk of orthologous strains [6] such as monkeypox virus (MPXV) and specific strains of the cowpox species (CPXV) [7, 8], as well as their potential use as biological weapon [9]. It is because of the latter that certain professional groups, including military personnel are still getting vaccinated.

The vaccinia virus smallpox vaccine used in the eradication campaign was highly effective, but was associated with adverse side effects. The frequency of side effects was acceptable at the time where smallpox was a major health threat but is unacceptable in the 21^st^ century. More recently a vaccinia virus clonal isolate, ACAM2000 has been used, produced using modern cell cultures [10–12]. Highly attenuated vaccinia strains such as Modified Vaccinia Ankara (MVA) have been available for decades [13]. Large MVA clinical trials, and clinical use, have found that MVA has an outstanding safety profile, but this is accompanied by a decreased immunogenicity resulting in the need for two immunizations. Moreover, since MVA usage was predominantly after smallpox eradication, the protective efficiency of MVA toward variola virus was not proven. Research on new orthopox vaccines continues, both in response to these concerns and as a means of understanding why the vaccinia virus smallpox vaccine is so effective.

The strategies leading to today's next generation candidate smallpox vaccines are diverse: they include, but are not limited to, the use of (i) vaccinia immunization in the presence of a small molecule inhibitor [14], (ii) DNA immunization using select immunodominant antigens [15–18], and (iii) soluble poxvirus proteins [19–21]. A limited number of immunodominant antigens [22] have been linked to successful protection: intracellular mature virion (IMV) antigens A27 [23, 24], D8 [25, 26], F9 [27], H3 [20, 28], L1 [29] and extracellular enveloped virion (EEV) antigens A33 [30–32], and B5 [33, 34]. Maximal protection is obtained with vaccines combining recombinant membrane proteins from both forms of the infectious virus (IMV and the EEV). The virus encodes the seven proteins A33, A34, A36, A56, B5, F12, and F13, that are specific for the EEV membrane [32, 34–38].

Among those, A33 is a 23 kDa, homodimeric type II transmembrane that undergoes both O- and N-glycosylation (N125 and N135) [32, 36, 39]. Both N-linked glycosylation sites are used in vaccinia but variola virus and monkeypox virus lack the equivalent N125 site [40]. A33 controls the incorporation of A36 into the EEV particle and subsequently the production of actin tails. Therefore, A33 plays an important role in effective cell-to-cell spread within the host [41–43]. A33 is also required for proper trafficking of B5 to the EEV-specific membrane and proper formation of infectious EEV [43, 44]. A33 contains a membrane-proximal cysteine on the A33 ectodomain that forms an intermolecular disulfide bridge. However, this cysteine is not required for the production of infectious extracellular virus [45]. The crystal structure of the ectodomain of A33 revealed an unusual C-type lectin-fold domain, similar in overall architecture to several NK cell ligands [40].

Antibodies targeting EEV proteins can protect against viral challenge. For example, anti-B5 mAbs are protective against lethal orthopoxvirus challenges in multiple small animals models [33, 46, 47] and anti-A33 antibodies can be protective in vivo [15, 30, 48, 49]. Protection mediated by anti-B5 antibodies is not mediated by conventional direct virus neutralization, as EEV is highly resistant to direct neutralization by anti-B5 antibodies, anti-A33 antibodies, and other EEV targets [46, 50, 51]. However, protective anti-B5 mAbs are highly efficient at facilitating complement-mediated neutralization of EEV, complement-mediated lysis of infected cells, and Fc-dependent protective mechanisms [46, 51, 52]. Mechanisms of protective action of A33 antibodies are similar to those of B5 [52].

Using X-ray crystallography, we mapped the A33 epitopes for three different antibodies and characterized their VACV-neutralization potential *in vivo*. We further identified one antibody (A27D7) that binds to A33 with high affinity, even when epitope residues are individually changed to other amino acids. We further tested the MAb binding to recombinant A33, where epitope residues were replaced with those from other orthopoxviruses and observed high affinity binding for A27D7. In an ectromelia virus (ECTV) infection model, A27D7 MAb was protective. In summary this suggests, that A27D7 is cross-species protective and able to neutralize a range of different orthopoxviruses *in vivo*.

## Results

### Monoclonal anti-A33 antibody characterization

We generated seven anti-A33 antibodies (A2C7, A26C7, A17D7, A25D11, A27D7, A25F2, and A20G2) by VACV immunization of mice followed by boosting with recombinant A33 protein. DNA sequencing revealed that the seven MAbs are assembled from five distinct heavy chain (HC) and 4 different light chain (LC) sequences (S1 Table): While A2C7, A17D7, and A25F2 have distinct HC sequences, A26C7 shares the HC with A27D7 and A25D11 shares the HC with A20G2, with only one amino acid exchange in CDR L1 (L to F). The LC sequences of A26C7 and A27D7 are nearly identical, while A25D11 is almost identical to A20G2. Both LC pairs only differ by one amino acid substitution in CDR1. A2C7 and A25F2 are derived from the same germline genes and are highly similar, while the A17D7 LC sequence could not be determined. In summary, antibodies A25D11 and A20G2 are identical except for one amino acid difference and the same is true for antibodies A26C7 and A27D7, resulting in a total of five distinct antibodies, of which we have complete sequences for A2C7, and A26C7/A27D7, A25D11/A20G2, and A25F2.

### *In vitro* A33 peptide binding and VACV neutralization

Peptide ELISA performed with 20mer peptides overlapping by 10 amino acids and covering nearly the entire amino acid sequence of A33, indicated that all anti-A33 MAbs bind only to recombinant A33 but not to any linear peptide (S1 Fig). Thus, we concluded that the seven MAbs recognized conformational epitopes on A33.

**Fig 1:**
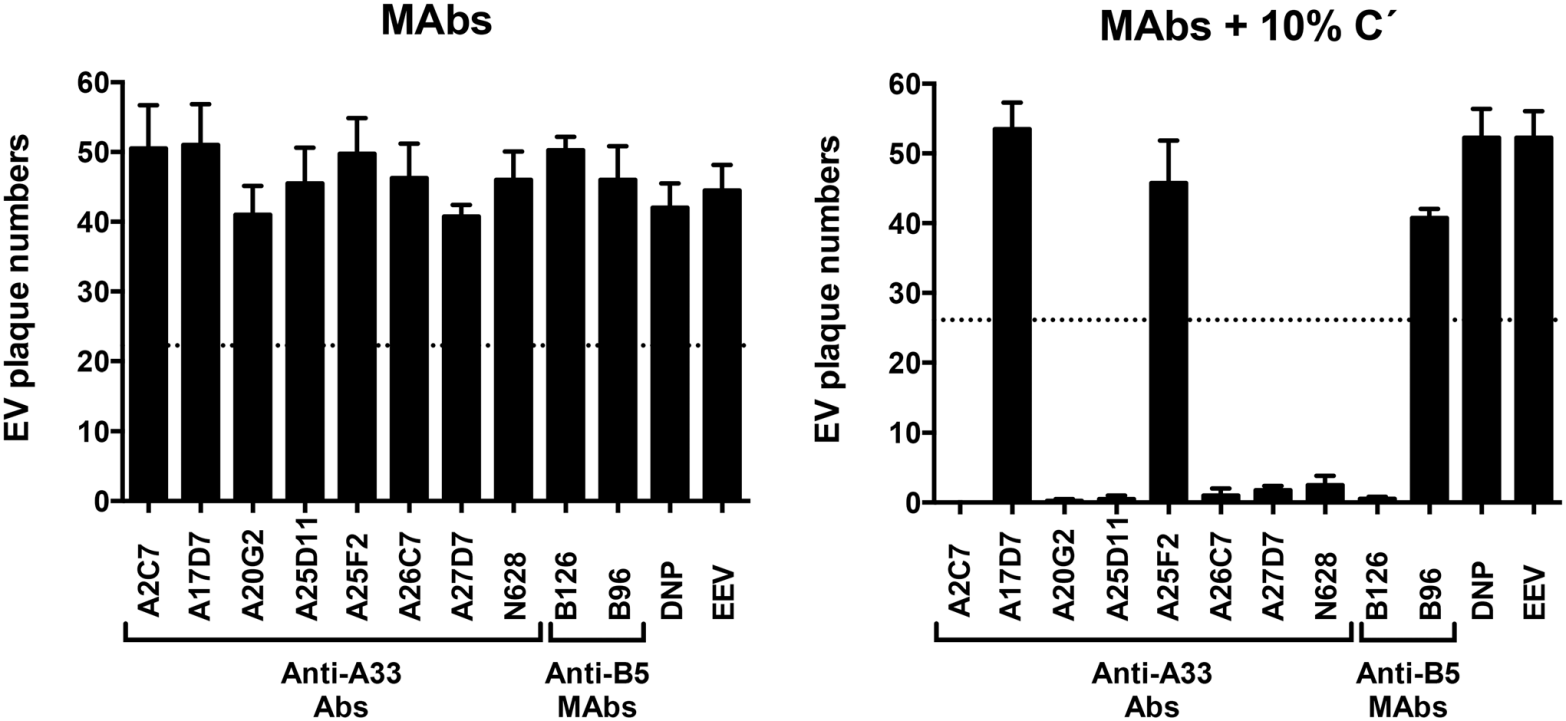
Complement and isotype dependence of anti-A33 MAb neutralization of VACV EEV. VACV EEV neutralization activity of purified anti-A33 MAbs in the absence (MAbs) or presence (MAbs+10% C´) of complement. Rabbit anti-A33 polyclonal Abs (N628) were used as positive control. Anti-B5 MAbs B126 (IgG2a), and B96 (IgG1) were used as positive and negative neutralization controls, respectively. Human anti-ditrophenol (DNP, IgG1) and VACV EEV (EEV) were used as negative controls. Error bars indicate SEM in each condition. Dashed line indicates the 50% of plaques number of VACV EV in panels A and B. The data are representative of two experiments. Three more experiments were done and they show comparable results.

All anti-A33 MAbs were then tested for their ability to neutralize EEV *in vitro* by complement dependent and independent processes (Fig 1). None of the antibodies was able to neutralize EEV in the absence of complement (Fig 1A). IgG1 anti-A33 mAbs A17D7 and A25F2 at 10 μg/ml exhibited no complement mediated EEV neutralization, consistent with the inability of recruiting complement by IgG1 MAbs in general (Fig 1B). However, IgG2a (A26C7, A25D11, A27D7, and A20G2) and IgG2b (A2C7) anti-A33 MAbs at 10 μg/ml exhibited strong complement-mediated EEV neutralization in the presence of complement (Fig 1B). Anti-B5 MAbs B126 (IgG2a) at the same concentration show comparable neutralization activity as anti-A33 mAbs of appropriate isotypes. Irrelevant IgG1 anti-DNP MAbs (DNP) and anti-B5 MAbs B96 (IgG1) at the same concentration showed no effect (Fig 1B). These data support a model where anti-A33 MAbs can neutralize EEV in the presence of complement via opsonization of the EEV particle surface.

#### A33-specific antibodies' ability to protect from an *in vivo* VACV challenge

**Fig 2:**
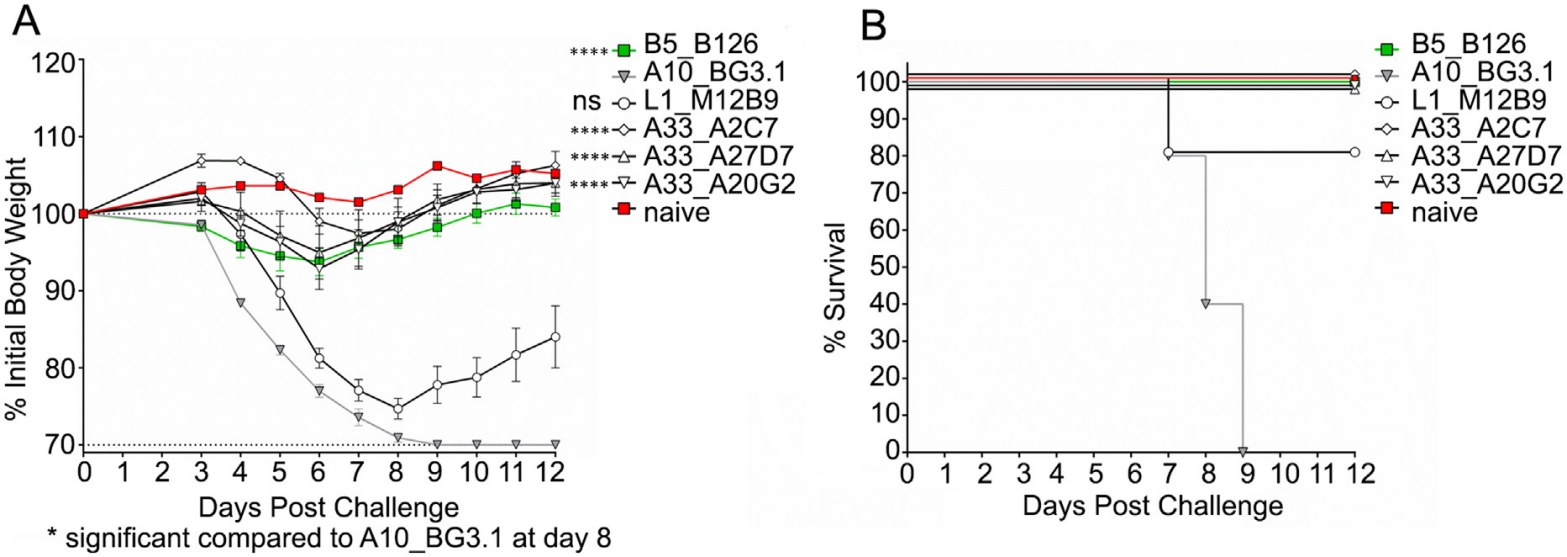
Protection of Balb/c mice from VACV_WR_ by anti-A33 mAbs. Antibody protection against weight loss (A) and mortality (B) caused by VACV_WR_ challenge. All analyzed anti-A33 MAbs protect both against weight loss and death. One of two independent experiments is shown. Significance for all A33 antibodies (****) was P<0.0001, while the anti-L1 control antibody M12B9 was not significant (ns) with P = 0.1577. The negative control antibody A10 provided no protection as expected, while the positive control anti-B5 antibody B126 conferred protection. (B) Note that slight shifts on the Y-axis were implemented for visualization purposes for all antibodies that confer full protection.

We next asked whether the three different neutralizing MAbs (A2C7, A27D7, and A20G2) were able to protect mice against an *in vivo* VACV challenge. Balb/c mice underwent intra-peritoneal injection of 100μg MAb on the day preceding infection (T = -1), followed by intranasal administration of 1x10^5^ PFU VACV-WR at D0 (Fig 2). Anti-B5 B126 MAb was used as a positive control. Anti-A10 BG3.1 MAb was selected as a negative control, since A10 is a core protein. Anti-L1 M12B9 MAb was added as a third control, with the expectation that it would protect against death but not weight loss, based on our previous experiments [53]. All tested anti-A33 MAbs showed outstanding protection against weight loss (Fig 2A) as well as death (Fig 2B). These results are consistent with our data on the *in vitro* neutralization experiments.

### Biochemical characterization of the A33/MAb interaction

**Fig 3:**
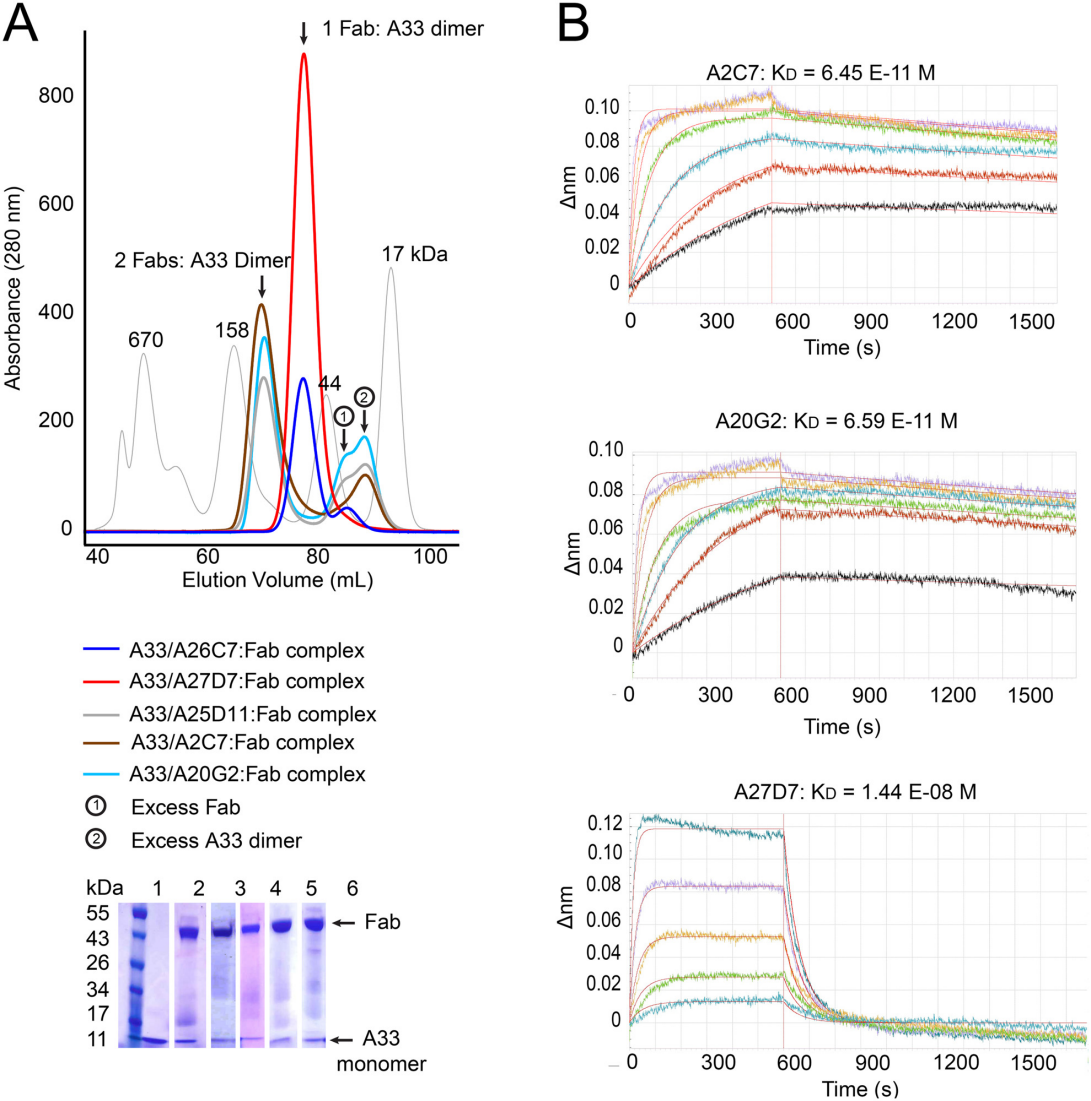
Fab/A33 binding interactions. (A) Determination of A33/Fab binding stoichiometry by size exclusion chromatography (SEC). Fab/A33 complex formation of five different MAbs (A2C7, A26C7, A25D11, A27D7, and A20G2) is illustrated. Two major peaks corresponding in size to 1 Fab: 1 A33 dimer (red and blue curve) and 2 Fabs: 1 A33 dimer (brown, cyan and grey) complexes are visible. Presence of both Fab and A33 in each peak are confirmed by SDS-PAGE (bottom left corner). Molecular weight markers with sizes in kDa are shown as a reference (thin grey curve). (B) Real-time A33 binding curves to immobilized MAbs as assessed by BLI. Note that A33 dissociates much slower from MAbs A2C7 and A20G2, compared to A27D7, likely to its ability to simultaneous bind to two Fabs.

To assess the interaction between the antibody and A33 we prepared the Fab portion of all five neutralizing antibodies (A2C7, A26C7, A25D11, A27D7, and A20G2) and assessed Fab binding to recombinant A33 using size exclusion chromatography (SEC) (Fig 3A). We observed two major peaks of different molecular weight (MW), indicating that the Fab’s bound to A33 with different stoichiometries. Both Fab’s A26C7 and A27D7 eluted with a lower MW when bound to A33, while the Fab’s of A2C7, A25D11, and A20G2 formed a higher MW complex with A33. The SEC data indicated that a single Fab of A26C7 and A27D7 bound to one A33 dimer, while A2C7, A25D11, and A20G2 each bound with one Fab to one A33 subunit (2 Fab’s per A33 dimer). We next assessed the real-time binding kinetics with the three representative antibodies A2C7, A27D7, and A20G2. Intact antibodies were immobilized on biosensors and binding to A33 in solution was measured by Biolayer interferometry (BLI) (S2 Table and Fig 3B). A33 bound with very high affinity to the antibodies A2C7 and A20G2 (K_D_ ~65 pM), consistent with the SEC data, where the A33 dimer can simultaneously bind to two separate Fab’s. This binding mode is characterized by a very slow dissociation rate. A33 also bound with high affinity to the antibody A27D7 (K_D_~14nM), yet roughly 400-fold weaker compared to A2C7 and A20G2. Since the A33 dissociation rates were very slow for both A2C7 and A20G2, which made calculation of the binding kinetics less robust, we repeated the binding assay but immobilized A33 instead of the MAbs (S2 Table). Both A2C7 and A20G2 showed similar binding to chip bound A33 in this reversed orientation with a ~2 to 4-fold difference in K_D_ each (S2 Table). However, MAb A27D7 showed very slow dissociation from A33, consistent with a model in which each Fab can bind to two separate A33 dimers on the sensor tip. As a result, the apparent binding affinity increased from 14 nM (MAb immobilized) to beyond 1pM (A33 immobilized), as no dissociation could be reliably measured. This led to A27D7 being the antibody with the highest affinity in this setting. All subsequent kinetic binding studies using A33 mutants were performed using A33 immobilized on the sensor tip. This binding orientation also mimics the antibody binding during infection, where A33 is embedded in the EEV membrane.

Of note, our recombinant A33 used for structural studies contained the three mutations Leu118Met, Lys123Ala, and Leu140Met that were previously incorporated and necessary for crystallization and structure determination [40]. We used the A33 variant Lys123Ala as “WT A33” as its mutation is outside the epitope of all tested MAbs (see below). When analyzed, the binding kinetics of the Leu118Met/Lys123Ala/Leu140Met mutant does not differ significantly from the WT surrogate (Lys123Ala), regardless of the choice of antibody (S2 Table).

### A33/Fab crystal structure determination and A33 epitope residue mutagenesis

**Fig 4:**
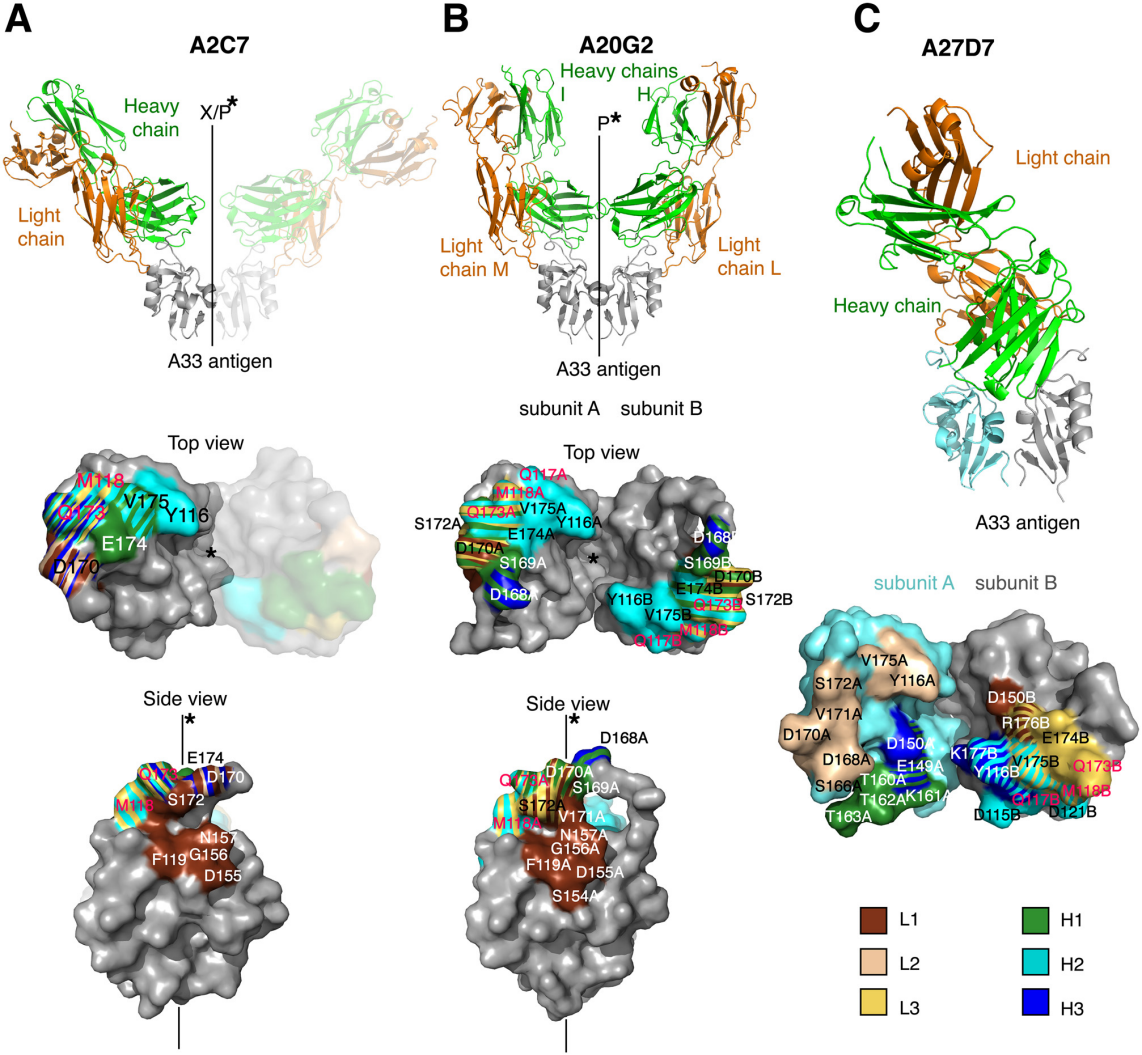
Structures of A33/A2C7, A20G2, and A27D7-Fab complexes. Structures of (A) A33/A2C7, (B) A20G2, and (C) A27D7-Fab complexes. Structures are shown as cartoon representations, with light chains in green and heavy chains in orange (top), while the antibody footprints on the molecular surface of A33 are shown below. In (C), A33 subunits (SUs) are shown in different colors because the epitope spreads asymmetrically over both SUs (SU A in aquamarine, SU B in grey). Footprints on A33 is colored according to contacting CDR loops (L1, brown; L3, yellow; H1, green; H2, cyan; H3, blue). Stripe patterns highlight A33 residues that elicit contacts with more than 1 CDR. The continuous line and the asterisk indicate A33 dimer axis. Letters indicate when this axis constitutes a crystallographic axis (X) along with the A33 dimerization axis (P). A33 residues that are part of the orthopox variation profile are labeled in magenta.

**Fig 5:**
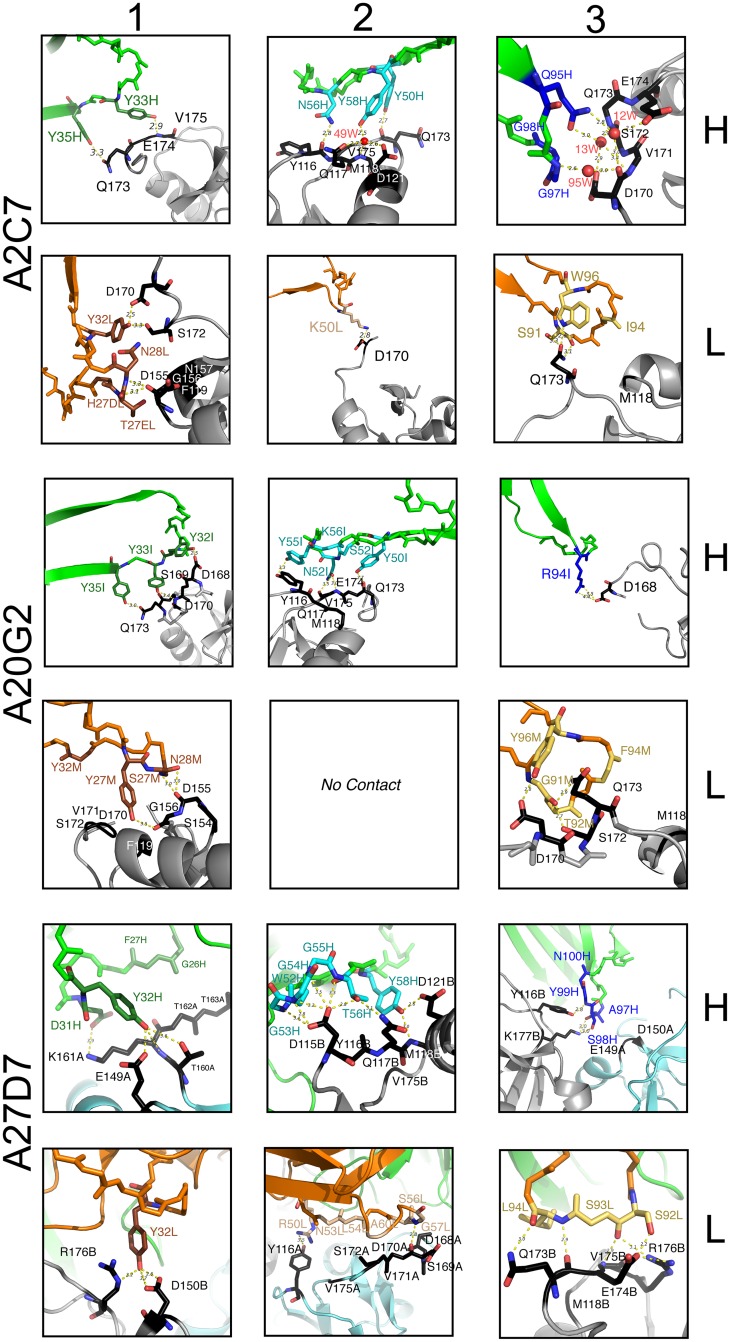
Detailed contacts at the Ag:Fab interface. Interactions between A33 residues and light-chain (L, orange) or heavy chain (H, green) CDRs (1–3) of the MAbs A2C7, A20G2, and A27D7. Residues that elicit actual contacts as defined in S3 Table are colored according to Fig 4. Main chain is shown uniformly for residues eliciting VdW interactions, while individual atoms are colored for residues eliciting electrostatic interactions. Amino acid side chains are shown when relevant. A33 backbone in grey, and contact residues in black. Only main chain atoms are shown when residues are involved in VdW interactions.

**Fig 6:**
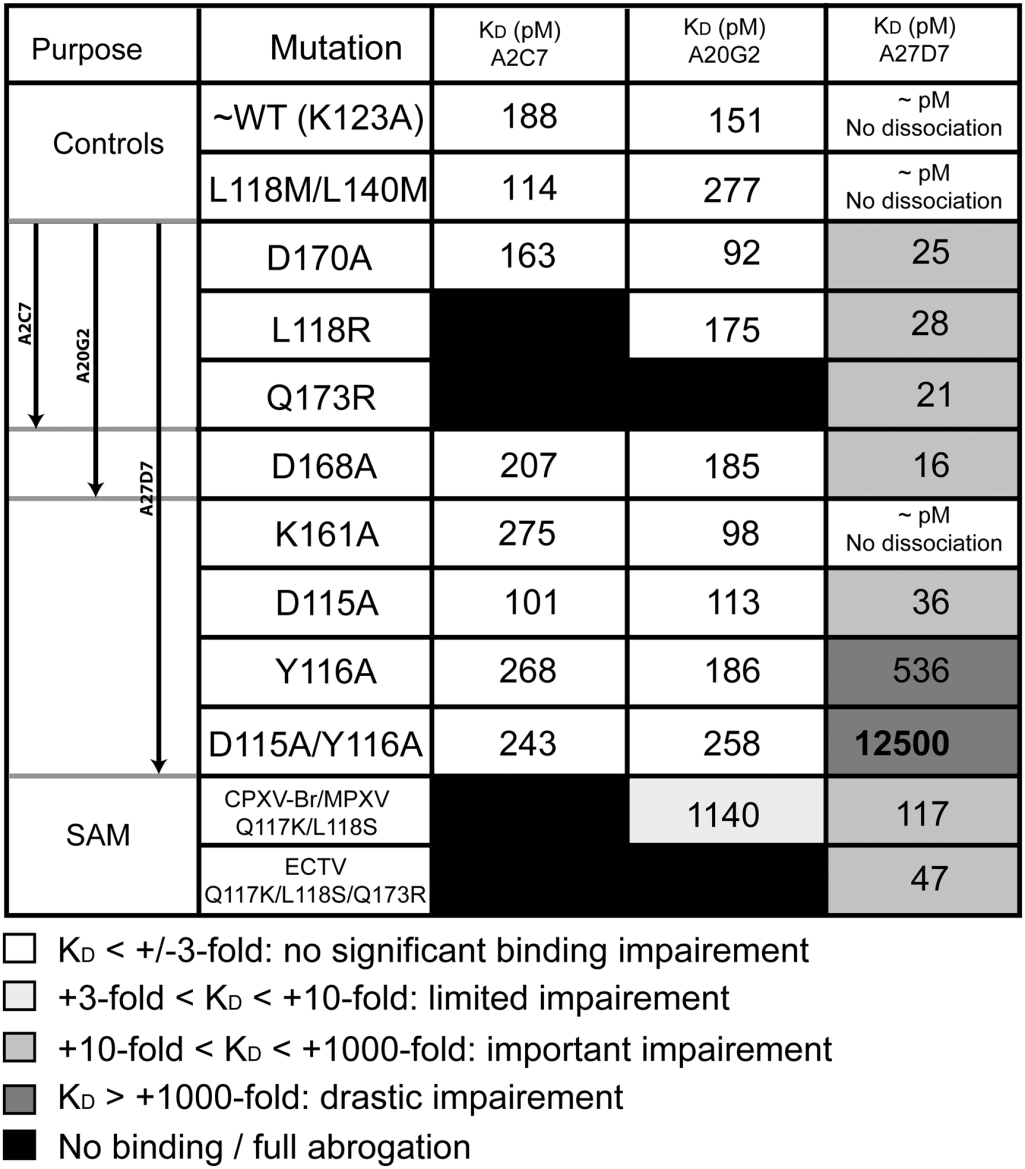
Binding kinetics of WT-A33 and mutants to antibodies A2C7, A20G2, and A27D7. Apparent equilibrium binding constants (K_D_) calculated from the binding curves shown in S3 Fig. Note that K_D_ values can include avidity effects, since both antibodies and A33 are dimeric proteins. Grey shading qualitatively illustrates the extent of binding impairment by the indicated A33 mutation (from white, no impairment to black, full binding abrogation). Lys123Ala (K123A) was required for successful crystallization and is considered as wild-type A33 (WT) since it is located outside any MAb epitope [40].

To analyze the binding mode of each Fab to A33 we set out to determine the crystal structure of one representative of each of the three different neutralizing MAbs bound to A33. To increase our chances of success we started out with all five neutralizing antibodies and finally determined the crystal structures of the complexes of A33/A2C7, A33/A20G2, and A33/A27D7 to 2.3 Å, 2.9 Å, and 1.6 Å resolution, respectively (Table 1 and Figs 4 and 5). To assess the impact of A33 epitope residues in antibody binding, we have further generated A33 variants with selected mutations in the epitope of each of the three crystallized antibodies and determined the antibody binding affinity using BLI (S2 Table and Fig 6 and S3 Fig)

**Table 1:** Data collection and refinement statistics of A33/Fab complexes.

**Data collection statistics**	**A33/A2C7**	**A33/A20G2**	**A33/A27D7**
PDB ID	4LQF	4LU5	4M1G
Space group	C222_1_	P2_1_2_1_2_1_	P3_2_21
Cell dimension			
a, *b*, *c*, (Å)	71.3, 73.2, 221.6	49.9, 157.3, 175.9	95.0, 95.0, 127.3
α, β, γ (°)	90, 90, 90	90, 90, 90	90, 90, 120
Resolution range (Å) [outer shell]	51.1–2.3 [2.42–2.30]	54.9–2.9 [3.06–2.90]	47.5–1.6 [1.69–1.60]
No. of reflections	25343 [3407]	30937 [4226]	87767 [12724]
R_merge_ (%)	9.5 [64.4]	22.8 [75.1]	8.2 [65.2]
Multiplicity	4.8 [4.4]	5.2 [4.9]	6.1 [6.3]
Average I/σI	11.3 [2.0]	5.8 [2.0]	13.9 [2.9]
Completeness (%)	96.9 [90.9]	97.8 [93.5]	99.3 [99.8]
**Refinement statistics**			
No. atoms	3990	8008	5015
Protein	3905	7949	4522
Waters	81	59	483
Inorganic	4	0	10
Ramachandran plot (%)			
Favored	98.4	95.2	98.1
Allowed	100.0	99.8	100.0
R.m.s. deviations			
Bonds (Å)	0.005	0.005	0.005
Angles (°)	1.01	0.98	1.06
B-factors (Å^2^)			
Protein	35.0	37.9	15.7
Waters	33.4	15.7	23.9
Inorganic	43.8	/	38.9
R factor (%)	19.7	23.4	20.1
R_free_ (%)	23.0	28.2	22.6

### The A33/A2C7 complex

The A33/A2C7 complex shows two Fab molecules symmetrically bound to the A33 dimer, consistent with our SEC data (Fig 3A). Interestingly, the asymmetric unit (ASU) of the crystal contained half of the biological assembly (A2C7 Fab bound to one A33 monomer), which dimerized along a crystallographic symmetry axis. Each A2C7- Fab molecule elicits, therefore, identical contacts with a discontinuous and conformational epitope at the membrane-distal extremity of each A33 subunit (Fig 4). The buried surface area (BSA) between A33 and the LC is 365.1 Å^2^, while the HC buries a total of 334.1 Å^2^ between A33, leading to a total BSA of 699.2 Å^2^ between Fab and A33, which corresponds to 4.9% of total protein surfaces (S4 Table). This is considerably smaller than the typical range found in antibody/ protein antigen complexes (1400–1700 Å^2^ BSA, [54]). The epitope of A2C7 appears unusually small, yet it contains eleven A33 residues that are in contact with sixteen residues from the antibody, eight from each chain. In contrast to other available VACV antigen antibody complex structures, including D8/LA5 [50, 55], L1/7D11 [56] and L1/M12B9 [53], the LC appears to be as important in antigen binding as the HC.

Shape correlation (Sc) measures the geometric surface complementarity of protein-protein interfaces and reflects their specificity [57]. Both heavy and light chains appear to bind with high specificity to the antigen (Sc = 0.67 and 068, respectively). For antigen-antibody interfaces, Sc values of 0.64 to 0.68 [58] have been reported, illustrating that the A2C7/A33 interaction is very specific. This interface is held together by an extensive network of hydrogen bonds and salt bridges involving every CDR loop (Fig 5). Additionally, a number of water molecules mediate H-bonds between A33 and A2C7 (S2 Fig). The presence of water molecules in the heavy chain interface may explain the slightly lower specificity observed for this chain, reflected by its Sc value of 0.67 vs. 0.68 for the light chain, since waters were not included in Sc calculations. Gln173 is a major contact residue of the A2C7 epitope and intersects with of almost every CDR loop. It forms multiple hydrogen bonds via its backbone and side chain (H1, H2, H3, and L3; Fig 5): The Gln173 backbone carbonyl elicits a favorable hydrogen bond with H2:Tyr50 hydroxyl, while the side chain amide group elicits a hydrogen bond with H1:Tyr35 hydroxyl. Additionally, its side chain amide group elicits favorable hydrogen bonds L3:Ser91 and with L3:Trp96. Because its backbone is involved in the interaction, we mutated this residue to an arginine instead of an alanine, on the basis that the bulky and positively charged side chain may induce steric clashes at the interface in the mutant and will therefore result in a decreased affinity. As expected, we observed complete loss of A2C7 binding to the A33 Gln173Arg mutant (S2 Table and Fig 6 and S3 Fig). Interestingly, the Gln173Arg substitution is found in ECTV suggesting that A2C7 would be unable to protect against ectromelia challenge. A33 residue Asp170 is located on a flexible loop and is contacted by both H and L chain. It forms a salt bridge with L2:Lys55 and a hydrogen bond with L1:Tyr32. However, the Asp170Ala mutation did not affect the binding affinity of A2C7-MAb, suggesting it is not a critical binding residue. It should be noted however, that for unknown reasons, the overall binding signal is much lower compared to the wild-type A33 (S2 Table and S3 Fig).

### The A33/A20G2 complex

The ASU of the A33:A20G2 complex contains 2 Fab molecules bound to one A33 dimer (1 Fab per A33 subunit). Although the epitopes on each A33 monomer should be identical, subtle differences have been observed between both binding interfaces and will be reported where necessary. This could be due to a subtle influence of crystal packing. A20G2 binds a similar epitope compared to A2C7. Sixteen residues of each A33 subunit are in contact with sixteen residues of the A20G2 Fab (S3 Table). Both H and L chain grasp the tip of each A33 subunit similar to a pincer, resulting in the binding of two Fab molecules per A33 dimer (Fig 4). The binding stoichiometry is consistent with the SEC data (Fig 3). The Sc value is 0.68 and 0.69 for both antibody/antigen binding interfaces, while the BSA is 802 and 864 Å^2^ for each of the two Fab/A33 interfaces (S4 Table). As such, the A20G2 epitope is slightly larger compared to A2C7. Despite numerous amino acid differences in the CDRs, A20G2 approaches the antigen with an overall similar topology compared to A2C7, with the exception of CDRs L2 and H3. This is reflected in an overall change in binding angle (Fig 4A and 4B). L1 plays a major role in both complexes, as it clamps onto one side of each A33 subunit (A33 residues Phe119, Asp155, Gly156, Asn157, Asp170, and Ser172, while Ser154 and Asn157 are exclusive to A20G2). L3, H1 and H2 target a similar epitope at the top outward edges of A33, centered around residue Gln173 in both A33 molecules. Similar to A2C7, the Gln173Arg mutation also fully abrogates A20G2 binding (Fig 6). In A2C7, the A33:Asp170-L2:Lys50 salt bridge is replaced against a hydrogen bond in A20G2 involving L3:Tyr96 (Fig 5). This is the result of L2:Lys50 in A20G2 being replaced with L2:Leu50 in A2C7. However, the Asp170Ala mutation did not have any impact on binding of A20G2 to A33, similarly to the binding of A2C7.

To compare the overall binding of A2C7 and A20G2 on A33, we superimposed both the variable regions of the L as well as H chains separately. The L chain superimposed well even at the CDRs (RMSD = 1.12 Å for Cα residues 1–119). However, heavy chains did not superimpose well overall (RMSD = 3.73 Å for Cα residues 1–115) but appeared to have a high structural homology when superimposing only residues until H3 (RMSD = 0.77 Å for Cα 2–94). We conclude that sequence divergences of these two antibodies had little effect over most of the antigenic interface, but that it induced major local topological differences in the CDR H3 region. This is not surprising since the H3 loop is much longer in A2C7 due to a four amino acid insertion (H3:ARQWGGAMDY), compared to A20G2 (H3:ARGMDY).

Interestingly, the A33 mutation Leu118Arg that is located on both epitopes for A2C7 and A20G2 had no effect on the A20G2 binding kinetics, while they fully impaired A2C7, likely due to a clash with L3, which approached A33 from a different angle. Similarly, the mutation Asp168Ala also did not impair binding of A20G2 significantly (S2 Table and Fig 6 and S3 Fig). In conclusion, A2C7 and A20G2 target similar but not identical epitopes mostly due to sequence differences within H3 and L3.

### The A33/A27D7 complex

A27D7 MAb engages A33 in a drastically different manner compared to A2C7 and A20G2. A single A27D7-Fab binds at the A33 dimer interface, with the L and H chains contacting both A33 subunits. This correlates well with the SEC data, which suggested that one Fab binds one A33 dimer (Fig 3). BSA for both L and H chains bound to A33 are larger compared to A2C7 and A20G2 (L = 525.3 vs. H = 630.2 Å^2^) for a total of 1155.5 Å^2^ (Fab:Ag) (S4 Table). Sc values of 0.71 and 0.74 are among the highest Sc values reported for any Fab:Ag interface and indicate a highly specific binding interaction.

Eighteen residues of the H chain contact sixteen A33 residues, while fifteen residues of the L chain contact twelve A33 residues (S3 Table and Fig 4). CDR H1 interacts only with A33 subunit A (Fig 4). H1:Tyr32 forms hydrogen bonds with three A33 residues (Glu149, Thr160, and Lys161), the most favored of which is with Glu149 (2.53 Å). In addition, a salt bridge is formed between Lys161 and H1:Asp31. Surprisingly, the Lys161Ala mutation did not impair A27D7 binding (S2 Table and Fig 6 and S3 Fig). CDR H2 interacts only with A33 subunit B. A33 residue Asp115 is a central residue of the H2 interface, as it forms six potential hydrogen bonds with H2 residues Gly53, Gly54, Gly55, and Thr56. Interestingly, the Asp115Ala mutation only slightly affects A33 binding (S2 Table and Fig 6 and S3 Fig). Three other hydrogen bonds link H2 residues Thr56 and Tyr58 to Gln117, Met118, and Asp121. Van der Waals contacts are the most extensive of the interface, with forty-two contacts compared to only eighteen in the H1/A33 interface. Water molecules do not contribute largely to this interface (S2 Fig).

CDR H3 is centrally positioned in the A33 dimer groove, and contacts residues of both A33 subunits. H3 forms three distinct hydrogen bonds with A33 subunit B: Lys177 interacts with H3:Ala97 and H3:Ser98. The third hydrogen bond is formed between Tyr116 and H3:Tyr99. Water molecules are recruited to the interface and allow for water-mediated hydrogen bonds with A33 subunit A residues Glu149 and Asp150. The H3 loop has a number of VdW contacts that is comparable to H1 (20 vs. 18). Overall, H3 appears to make a modest contribution to the interface compared to H1 and H2.

The A27D7 light chain does not elicit any salt bridge with A33, and its interaction with the antigen is mostly driven by van der Waals contacts. In particular L2 and L3 each interact specifically with either one of the A33 subunits. L1 is relatively short compared to group II MAbs A20G2 and A2C7 (12 vs. 16 residues). A single residue L1:Tyr32, forms one hydrogen bond with Asp150 and also accounts for all VdW contacts with the A33 subunit B. L2 interacts solely with A33 subunit A, with a total of two hydrogen bonds and 30 VdW contacts. Moreover, four residues that elicited VdW interactions are involved in a water-mediated hydrogen bond network between L2 residues Asn53, Leu54, Ser56, and Ala60 of A27D7 and A33 residues Gln173_A_, Asp168_A_, Ser172_A_, and Val175_A_. CDR L3 (Ser92 and Leu94) forms an extensive hydrogen bond network with A33 residues Gln173_B_, Glu174_B_, Val175_B_ and Arg176_B_. Binding affinity of A27D7-MAb to A33 mutant Gln173Arg, however, remained in the picomolar range (K_D_ = 20 pM), suggesting that the many contacts formed between A27D7 and A33 can compensate for the various single amino acid substitutions on A33 (S2 Table and Fig 6 and S3 Fig).

As the Fab of A27D7 does not form identical contacts with both subunits of A33, some A33 residues will only be in contact with the Fab once. However, Tyr116, Asp150, and Val175 form contacts with the Fab in both A33 subunits, and among those, Tyr116 was chosen for single alanine scanning mutagenesis, as it might contribute more to the overall binding energy than the previously assessed A33 residues (Fig 6). The Tyr116Ala mutation led to a significant decrease in binding affinity (S2 Table and Fig 6 and S3 Fig; K_D_ = 0.54 nM). Next, we asked whether the moderately reduced binding affinity of the mutants Asp115Ala, Lys161Ala, Asp170Ala and Gln173Arg could be the result of a particularly mutation-tolerant A27D7/A33 binding interface, or because these residues are not making important contacts with the antibody. To answer this question, we created the double mutant Asp115Ala/Tyr116Ala and observed a synergistic effect of the mutations, as affinity decreased another ~20-fold compared to Tyr116Ala only (S2 Table and Fig 6 and S3 Fig; K_D_ = 12.47 nM). However, even combining both mutations still resulted in low nM binding affinity suggesting that a large number of energetically favorable interactions still remain intact and enable the specific binding interaction.

### Cross-species neutralization abilities of anti-A33 antibodies

The robustness of A27D7 binding to single alanine A33 mutants made this antibody an attractive candidate for cross-neutralization studies. We therefore explored the potential of our anti-A33 (VACV) MAbs to bind to recombinant A33 that mimics the epitope of the orthopox strains such as cowpox Brighton (CPXV-Br), MPXV, and ECTV. Sequence alignment of A33 from different orthopoxviruses revealed that no more than three residues out of the thirty-eight possible variations differ in the A33 epitopes among the analyzed orthopox strains (S4 Fig). The A2C7 epitope contains only two of those variations, located at positions 118 and 173. A27D7 and A20G2 epitopes contain the same, plus an additional residue at position 117. All residues that may vary within the epitopes are highlighted in red (Fig 4), regardless of the viral strain. Using site-directed mutagenesis we made two recombinant forms of A33. The A33 variant Gln117Lys/Leu118Ser both mimics the epitope of CPXV and MPXV, while the A33 variant Gln117Lys/Leu118Ser/Gln173Arg is specific for ECTV. Both antibodies A2C7 and A20G2 failed to bind to the ECTV variant, while A20G2 still bound with a ~9-fold lower affinity to the CPXV/MPXV variant. As expected however, A27D7 was able to bind both A33 variants with high affinity (K_D_ of 47–117 pM) (S2 Table and Fig 6 and S3 Fig). Therefore, we next asked whether A27D7 could protect against ectromelia infection, since ectromelia A33 contains the most amino acid changes in the A27D7 epitope compared to the other orthpox species.

### ECTV challenge

**Fig 7:**
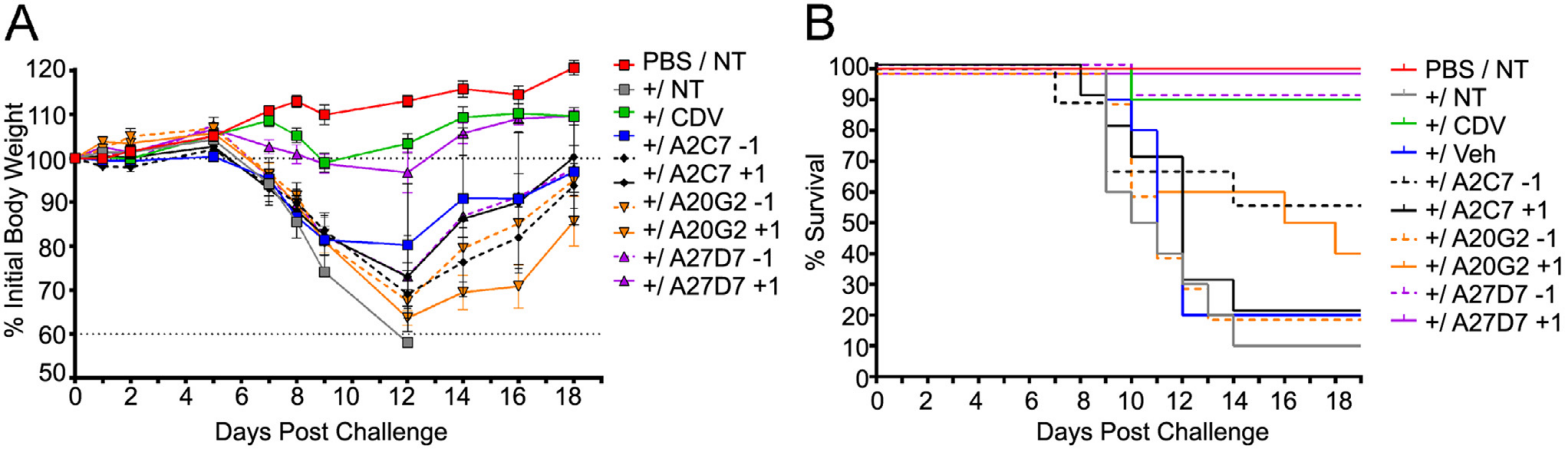
Treatment with antibody A27D7 protects mice against a lethal ECTV challenge. Mice were treated with A2C7, A20G2 or A27D7 at T = -1 or T = +1 relative to a 1000 PFU ECTV challenge. (A) Mice were monitored for morbidity, as measured by weight-change. Mice treated with A27D7 at T = +1 were fully protected against morbidity. N = 5 mice/group. Data presented is from 1 of 2 studies—both with similar results. (B) Mice were monitored for mortality until T = +21. Only mice treated with A27D7 were significantly protected against challenge. P = 0.0004 for T = +1 and P = 0.006 for T = -1 (90% protected). N = 10 animals/group. Data is pooled from 2 independent studies of 5 animals/group. Controls include no ECTV challenge (PBS/NT), challenge without treatment (NT), treatment with vehicle only (Veh) or CDV treatment.

In order to demonstrate the in vivo protective capabilities of antibodies A2C7, A27D7, and A20G2 we evaluated their efficacy following a lethal challenge with ECTV. Mice were treated with a single 100 μg IP injection of the antibodies at either T = -1 or T = +1 relative to challenge with 1000 PFU of ECTV. An additional group of mice was treated with the potent antiviral cidofivir (CDV), which is known to be protective against this dose of ECTV. Following challenge, we observed that mice treated with antibodies A2C7 and A20G2 were not able to statistically protect mice against challenge when compared to mice treated with vehicle (Fig 7A). Conversely, mice treated with the A27D7 antibody were fully protected against challenge when the antibody was administered at T = +1 (P = 0.0004) and were 90% protected (P = 0.006) when the antibody was administered at T = -1 (Fig 7A). Although weight-loss was significant following the T = -1 administration, we found negligible levels of weight-loss when the antibody was administered at T = +1 (Fig 7A). Indeed, at T = +1 we found that A27D7 protected against mortality and morbidity with the equivalency of the CDV-treated controls (Fig 7B). The slightly reduced efficacy of the antibodies when administered at T = -1 is likely a reflection of the antibody half-life, which we have not addressed in this assay. At T = +21 days post-challenge, we re-challenged the mice with a 10,000 PFU inoculum of ECTV—all previously challenged mice were protected against subsequent mortality and morbidity, indicating that the antibodies did not impede the generation of memory and protective immunity.

## Discussion

In this study, we have generated a panel of anti-A33 MAbs and characterized their binding to recombinant A33, as well as assessed their complement-dependent neutralization activity of VACV EEV. The three neutralizing MAbs, A2C7, A20G2, and A27D7 were further tested in a VACV_WR_ challenge model, where they all demonstrated full protection. For each of these antibodies we determined the A33 epitope using X-ray crystallography and assessed the robustness of antibody binding to A33 variants with amino acid exchanges in the various epitopes. We further identified that A27D7 was unique in its binding to A33 and exhibited resistance to single alanine substitutions within the epitope. A27D7 further bound to engineered A33 mimicking other orthopox strains within its epitope and was protective against in vivo challenge with ECTV. In contrast, both A2C7 and A20G2, which failed to bind the ECTV A33 mimic failed to protect against ECTV challenge as expected. The strong protection of A27D7 against ECTV challenge at day 1 post infection is surprising but we believe a reflection of its unique binding to A33, in which the antibody-antigen interface can tolerate several amino acid exchanges and still bind with high affinity. There is slightly reduced protection when the A27D7 is administered 1 day prior to ECTV infection. As A27D7 fully protects against VACV when given at day -1, it could be that the antibody half-life *in vivo* is more important during the ECTV challenge, especially since the A27D7 binds with reduced affinity to the ECTV A33 mimic compared to VACV A33 and a higher dose of antibody could therefore be needed for full protection.

A former study showed that VACV-A33 vaccination protected mice against ECTV but interestingly not against CPXV-Br [31]. The reason for the lack of protection remains unclear, because VACV is more closely related to CPXV-Br than to ECTV (S4 Fig). To address this conundrum, we have developed an *in vitro* assay to assess the potential cross-neutralization ability of a given antibody in three steps. First, we used single alanine mutagenesis to engineer variants of A33 in which epitope residues that were predicted to be important for antibody binding based on our structural data were targeted. Next, the real-time binding kinetics of the A33 variants to the different antibodies was tested. Antibodies that showed robust binding to different A33 variants (e.g. A27D7) were further assessed in their ability to bind to A33 variants that have been engineered in their epitope to mimic the sequence of other orthopox strains, including ECTV and CPXV/MPXV. If high affinity binding to these A33 cross-species variants is still observed, we predict that the antibodies would be protective. Using such *in vitro* approach, rather than expressing all of the orthologous A33 antigens individually, is highly time and cost efficient and sufficient to predict the outcome of specific MAb-guided protection experiments, and does not require any stringent safety procedure associated to virulent strains.

In our experiments the antibody A2C7, which failed to bind to the A33 Gln173Arg ECTV mimic, also failed to protect against lethal challenge with ECTV, as expected. Position 173 is occupied by arginine in ECTV, while glutamine is conserved in all other strains (S4 Fig). Position 173 is also common to all three epitopes and we expected Gln173Arg mutation to decrease binding of all three MAbs. It indeed resulted in abrogating A33 binding to A2C7 and A20G2. However, its effect was opposite in A27D7, as it reversed the loss of binding conferred by Gln117Lys/Leu118Ser mutations. Beside favoring A27D7 in terms of protectivity for the aforementioned reasons, our study also illustrates how divergent the properties of antibodies targeting a given Ag can be. In this study, we have ranked our representative MAbs using various properties, such as binding affinity the location and composition of the epitope, the binding stoichiometry (1 or 2 Fab per A33 dimer), and the tolerance to alanine scanning mutagenesis within the epitope. The most potent antibody (highest affinity) is not necessarily the top candidate in each category and each MAb may be raised and optimized for a particular property. For example, A27D7 is characterized by a >10-fold higher binding affinity for VACV-A33 compared to A2C7 and A20G2. However, A2C7 and A20G2 present specific binding properties, such as increased MAb clustering resulting from A33 bivalency. This suggests that when considering a particular antigen, the efficiency of an antibody response most likely relies on the combination and the synergetic effect of different antibodies targeting a specific Ag. This added layer of complexity has been so far overlooked, and we hope that its study will be implemented in future protection models. This could be studied in an *in vivo* protectivity assay by comparing the responses obtained while using individual MAbs, to those obtained using cocktails combining MAbs with different features, such as discussed above.

The data we obtained using single alanine scanning mutagenesis within the A27D7 antibody epitope may explain why a previous study observed that vaccination with recombinant Sindbis virus expressing A33 VACV conferred protection against ECTV but not CPXV [31]. In this case, vaccination might not have elicited anti-A33 antibodies that could bind the Gln173Arg variation found in cowpox. Residue 173 is adjacent in the A33 crystal structure to residues 117 and 118 and as such topographically part of the CPII region that the authors identified as containing the protective epitope. We observed a similar effect for A27D7, which bound tighter to our A33 ECTV mimic than to the CPXV counterpart, after mutating this single residue from a Gln to an Arg. However, while we have not tested if A27D7 can protect against cowpox challenge, binding of the MAb to the cowpox A33 epitope variant is still in the nanomolar range, which we predict to be sufficient to confer protection.

## Materials and Methods

### A33 protein expression and mutagenesis

The A33 expression vector (VACV strain Acam2000 GenBank: AY313847) containing the two mutations Leu118Met and Leu140Met was graciously provided by David Garboczi (NIH/NIAID). Recombinant A33 protein (residues 89–185) was produced and purified as previously described [40]. Briefly, protein was expressed in BL21 CodonPlus *E*. *coli* cells (Agilent) and refolded by rapid dilution. Protein was then purified by Ni-NTA affinity chromatography using a 5 mL HisTrap FF column (GE Healthcare) with bound protein eluted in 0.2 M Imidazole, 20 mM Tris pH 8.0, 0.3 M NaCl. The eluted protein was then further purified by gel filtration chromatography on a Superdex 200 size exclusion column (GE Healthcare). Pure A33 eluted at a volume corresponding to a dimer. A biotinylated version of A33 (BtA33) was engineered by fusing an Avitag to the N-terminus of A33 and recombinant A33 was enzymatically biotinylated according to manufacturers suggestion (Avidity).

BtA33 mutants were engineered by site-directed mutagenesis using the Quickchange II mutgenesis kit and verified by sequencing. Engineered mutations include the single mutants Lys123Ala, Leu118Arg, Tyr116Ala, Asp170Ala, Gln173Arg, Asp168Ala, Asp115Ala, Lys161Ala, the double mutants Asp115Ala/Tyr116Ala, Gln117Lys/Leu118Ser and the triple mutant Gln117Lys/Leu118Ser/Gln173Arg.

### Generation of anti-A33 MAbs

For the generation of anti-A33 Mabs, BALB/C mice were infected intranasally with a sub-lethal dose (~0.5 x 10^3^ PFU) of VACV WR and subsequently boosted twice with intraperitoneal injection of ~100 μg of recombinant A33 protein. Hybridomas were generated from the immunized mice, and were screened for their specificity for VACV with an immunofluorescence assay, in which WR-infected HeLa cells were stained with culture supernatants of the hybridomas as described previously [59, 60]. Anti-B5 Mabs, (B126 or B96) were generated as described previously [46]. Rabbit-anti L1 sera were generated by immunizing rabbit with recombinant L1 protein as described in [61]. Rabbit anti-A33 polyclonal Abs (NR-628), pAbs, was produced by immunization of rabbits with recombinant A33R protein (rA33) and was obtained through the NIH Biodefense and Emerging Infections Research Resources Repository, NIAID, NIH (BEI Resources, Manassas, VA) from Drs. Eisenberg RJ and Cohen GH. [49].

### Gene sequences of heavy- and light-chain variable regions of Mabs

Total RNA from 300μL hybridoma cells in solution was isolated using the NucleoSpin RNA II kit according to manufacturer’s instructions (MACHEREY-NAGEL). cDNA was amplified using the OneStep RT-PCR kit (Qiagen). The reverse transcription PCR was performed using primers 5’MsVHE and 3’Cy2c outer (for isotype IgG2a antibodies A26C7, A25D11, A27D7, and A20G2) or 3’Cy2b outer (for isotype IgG2b antibody A2C7) for the heavy chains, and primers 5’mVkappa and 3’mCĸ for the kappa light chains [62]. The cycling profile was slightly modified from manufacturer’s recommendations and set up as follows: 1 cycle of 30 min at 50°C and 15 min at 95°C; 40 cycles of 30 s at 94°C, 45 s at 60°C (for heavy chains) / 58°C (for light chains), and 55 s at 72°C; followed by 1 cycle of 10 min at 72°C and a 12°C cool down. PCR products were verified by gel electrophoresis with a ~500bp product for heavy chains and ~450bp product for light chains. Afterwards, PCR products were purified using the QIAquick PCR Purification Kit (Qiagen) and subsequently sequenced by Retrogen (provided with the respective 5’ primer for heavy and light chains). Sequences include V-D-J regions for heavy chains and V-J regions for light chains. Finally, antibody germ lines were determined using IMGT’s V-Quest server [63].

Alternatively, Illumina MiSeq library prep was performed by standard Illumina methods. Briefly, the used Nextera XT DNA sample preparation kit uses an engineered transposome to fragment and tag (“tagment”) input DNA simultaneously, adding unique adapter sequences in the process. A limited-cycle PCR reaction using those adapter sequences was performed to amplify the inserted DNA. The PCR also adds index sequences on both ends of the DNA, thus enabling dual-indexed sequencing of pooled libraries on the Illumina MiSeq instrument to generate approximately 100.000–300.000 paired-end reads per sample. Data was then analyzed via IMGT/HighV-QUEST.

### Linear peptide ELISA

Biotinylated 20-mer peptides overlapping by 10 residues and covering nearly the entire amino acid sequence of A33 (residues 1–180 vs. 1–185) were synthesized by A&A Labs (San Diego, CA). Microtiter plates were coated with 100 μl of NeutrAvidin biotin binding protein (1 μg/ml) diluted in PBS overnight at 4°C (Thermo Scientific Pierce, Rockford, IL) and linear peptide ELISA for the purified anti-A33 Mabs was done as described previously [55]. 100 μl of recombinant A33 protein (A33) at 1 μg/ml in PBS was used to coat the plate as control. The secondary Ab was streptavidin-horseradish peroxidase-conjugated goat anti-mouse IgG (Invitrogen, CA).

### Viruses

MV of VACV_WR_ (Western Reserve) strain stock was grown in HeLa cells in D-10 (DMEM + 10% FCS + pen/strep/glutamine) as described previously [61]. Fetal calf serum (FCS) used in all experiments was heat inactivated (56°C, 30 min) prior to use to eliminate complement activity. Purified VACV_WR_ stock was made via centrifugation through a sucrose cushion as described previously [61]. Virus was stored at -80°C.

EEV stocks of VACV WR strain were prepared using HeLa cells grown in D-10, and the medium containing EEV was harvested at 2 days after infection as previously described [46] [50]. Clarified supernatant was used immediately or stored at 4–8°C for a maximum of 3–4 weeks. EEV VACV_WR_ stocks (~5 x 10^5^ PFU/ml) were untwined on VeroE6 cells in the presence of rabbit anti-L1 Abs to block contaminating MV or damaged EEV present in the EEV stock, as previously described [46] [50].

### EV neutralization

Briefly, VeroE6 cells were seeded at 1.5 x 10^5^ cells/well into 24-well Costar plates (Corning Inc, Corning, NY) and EEV VACV_WR_ neutralization was performed the following day using Mabs or pAbs samples at 10μg/ml (final concentration) or 1/100 (final dilution) respectively in the absence or the presence of baby rabbit complement 10% (final concentration) (complement, Cedarlane Laboratories, Ontario, Canada) as described previously [46] [50]. (Rabbit anti-L1 (1:25 to 1:100, final concentration) was used to block the MV present in the EEV stock in all experiments. VACV EEV supplemented with anti-L1 antibody was regularly used in each assay with or without complement as a negative control.

### Mouse intranasal VACV challenge

Female BALB/c mice were used at an age of 7–8 weeks. To infect the mice, a Pipetman was used to place 10 μl of VACV_WR_ on each nare of an isofluorane-anesthetized mouse (total volume, 20 μl), and the liquid was rapidly inhaled by the mouse. Mice were weighed daily to assess disease progression. Mice were euthanized if and when 25% weight loss occurred. A dose of 1x10^5^ PFU of VACV_WR_ was the standard dose given to 7-week-old BALB/c females. For A33 mAb protection studies, mice were treated by i.p. injection with 100 μg of antibodies 1 day before infection. Control mice received anti-A10 BG3.1 antibody, which is known to provide no protection. An additional group received anti-B5 B126 as positive control. A third group received anti-L1 M12B9, which was expected to protect against death but not weight loss [53].

### Fab preparation

A2C7 (mouse IgG2b), A27D7, and A20G2 (mouse IgG2a) Mabs were digested with 2% (wt/wt) activated papain for 4hr at 37°C in digestion buffer (A27D7: 50 mM NaOAc pH 5.5; A2C7 and A20G2: PBS pH 7.4). Papain was activated by incubating 24.4 μL papain solution at 20.5 mg/mL (Sigma) in 1 mL solution containing 100 μL 10X Papain Buffer (1M NaOAc pH 5.5, 12mM EDTA, and 10mM cysteine) for 15 min at 37°C. The papain digestion was inhibited with 1 mL of 200 mM iodoacetamide (IAA). A27D7 sample was then dialyzed in 2 L PBS pH 8.0 overnight at 4°C. For A2C7 and A20G2, papain reaction mixtures were diluted in one volume of PBS pH 8.0. All three samples were passed through a pre-equilibrated 1 mL protein A FF column in PBS pH 8.0 binding buffer. The purified Fab, contained in the protein A flowthrough, was concentrated to 1 mL using centrifugal filtration devices (Pierce Concentrator; 9-kDa molecular mass cut-off (MWCO)–Thermo Scientific) and purified to homogeneity by Size Exclusion Chromatography (SEC) on a Superdex 200 HiLoad 16/60 (GE) column in 20mM Tris pH 8.0, 200mM NaCl running buffer. All Fabs eluted as a single and sharp peak (V_e_ ~ 86 mL).

### A33:Fab complex preparation

A33 and Fabs were mixed together as homogeneous species at a molar ratio of 1:2 A33 dimer to Fab (A2C7 and A20G2) or 1:1 (A27D7) at a low concentration (< 1mg/mL). Each A33:Fab complex was concentrated to a volume of 1 mL using centrifugal filtration devices and SEC-purified (Superdex 200 HiLoad 16/60) in 20 mM Tris pH 8.0, 200 mM NaCl to separate the A33-Fab complex from unbound A33 and/or Fab. Fractions corresponding to the complex were pooled and concentrated for crystallization.

### Crystallization, X-ray data collection, and model building

#### A33/A2C7-Fab complex (PDB ID 4LQF)

Best crystals of A2C7-Fab/A33 complex were grown in 20% PEG3000, 0.1 M imidazole pH 8.0, 0.2 M zinc acetate with a protein concentration of 5.1 mg/ml. Structure was solved by molecular replacement (MR) from a single data set collected at SSRL beamline 11.1 and processed with Imosflm to the resolution of 2.3 Å [64]; Data was scaled and merged with Scala (ccp4i) [65],in space group C222. The asymmetric unit (ASU) of the crystal contained one Fab bound to one A33 monomer based on a Matthews coefficient of 2.41 Å^3^/Da a solvent content of 49.0% [66]. Molecular Replacement (MR) was performed with Phaser MR [67] using the A20G2-Fv domain (reported herein), the conserved domain of 3E5 IgG3 Fab (from pdb code 4HDI), and one monomer of A33 (from pdb code 3K7B). A single MR solution was found with space group C222_1_ (R = 52.4%). Model was refined using iterative cycles of model building in COOT [68] and restrained refinement using Refmac (ten cycles; CCP4i)[66]. Intensity-based twin refinement calculations were used throughout restrained refinement since Scala L test suggested partially twinned data (L = 0.475; untwined 0.5 vs. perfect twin 0.375) [69]. Isotropic Translation/Libration/Screw (TLS) refinement was also performed after partitioning every chain A, H, and L in five TLS groups according to the TLSMD server, as follows: A33 (chain A): 99–112, 113–135, 136–141, 143–175, 176–184; A2C7 chain H: 2–28, 29–63, 64–118, 119–179, 180–216; A2C7 chain L: 1–101, 102–114, 115–159, 160–177, 178–219. After including TLS refinement, we observed a reduction in R/R_free_ of 0.48/0.42% and an overall improvement in electron density, especially in the area of light chain residues R161-N167. After full refinement, model was ranked by Molprobity in the 99^th^ percentile for clashes and in the 100^th^ percentile for geometry, compared to structures of similar resolution, with a final R/Rfree of 19.70/23.0% [70].

#### A33/A20G2-Fab (PDB ID 4LU5)

Crystals grew in 20% PEG3350 (w/v), 8% tacsimate (v/v) pH 7.0 (vapor diffusion, sitting drop) with a protein concentration of 7.5 mg/ml. Data was collected up to 2.55 Å at SSRL BL11.1. Indexation and integration performed to 2.9 Å with Imosflm in P222. The MR starting model for the Fab portion of the complex [pdb code 1F8T], was selected based on the highest identity score in a PDB-BLAST. MR was performed using Phaser MR [67] and a single MR solution was found with space group P2_1_2_1_2_1_, with one A33 dimer and two Fabs occupying the asymmetric unit (R = 54.3%, Mathews coefficient of 2.83 Å^3^/Da and 56.5% solvent content). A first ten-cycle refinement with Refmac (including geometry weighting term of 0.005 and NCS restraints) gave an R/R_free_ of 33.3/37.4% [66]. Refinement was then performed alternating between manual refinement with COOT [68] and restrained refinement using Refmac [66]. NCS refinement was dropped at the last refinement stage. After full refinement, model was evaluated with, and ranked by Molprobity in the 100^th^ percentile for clashes and in the 99^th^ percentile for geometry, compared to structures of similar resolution, with a final R/R_free_ of 23.4/28.2% [70].

#### A33/A27D7-Fab (PDB ID 4M1G)

Initial crystals grew in 0.1 M sodium cacodylate pH 6.5, 0.2 M NaCl, 2.0 M ammonium sulfate with a protein concentration of 5.1 mg/ml (JCSG+ #50; 0.2+0.2 ul drops, vapor diffusion, sitting drops). Data from a single crystal was collected at SSRL BL11-1. MR was performed with Phaser MR [67] and a single MR solution was found with space group P322_1_ (R = 50.71%). The SEC profile of the complex suggested one Fab molecule bound to two A33 molecules (*i*.*e*. physiological A33 dimer), for a total MW of ~72 kDa resulting in a Matthews coefficient of 2.30 Å^3^/Da and 46.5% solvent. On this basis, MR was carried out with three independent components as starting models: the variable domain of the anti-tetrahydrocannabinol Lκ/HigG1 Fab fragment [pdb code 3LS4], its associated conserved domain, and one A33 monomer extracted from the previously deposited A33 dimer structure [pdb code 3K7B]. This Fab template shares the highest light chain identity (97%) with A27D7, among reported X-ray structures. Alternating cycles of manual refinement with COOT [68] and restrained refinement using Refmac [66] led to the final model (R/R_free_ = 20.1/22.6%), which ranked in the 99^th^ percentile for clashes and in the 100^th^ percentile for geometry, compared to structures of similar resolution (Molprobity) [70].

### Real-time binding kinetics by BioLayer Interferometry (BLI)

To assess binding kinetics of MAbs A2C7, A20G2, and A27D7 to WT-A33, we performed BLI assays using the OctetRED instrument (ForteBio, Inc). In a first experiment we immobilized MAbs on Anti-mouse Fc Capture (AMC) biosensors and tested binding to WT-A33 in solution. Biosensors were loaded with 10 μg/mL MAb diluted in 1X kinetics buffer (PBS pH 7.4, 0.002% Tween 20, 0.01% BSA) over 5 mins. MAb-loaded tips were tested against two-fold serial dilutions of WT-A33 analytes (0.78–50 nM). Binding kinetics were calculated using Octet Data Analysis 7.1 software (Forte Bio, Inc.) with curve-fitting statistics. The association steps were performed over 10 mins and dissociation steps performed over 20 mins. A negative control antibody (EE11) that targets the VACV antigen D8 was run in parallel to all assays for subtraction of background binding signal. Curves were aligned to baseline.

To test the effect of the A33 mutations on antibody binding, we immobilized biotinylated A33 (BtA33) using streptavidin (SA) biosensors. SA biosensors were loaded with 1.5 μg/mL BtA33 protein in 1X kinetics buffer (PBS pH 7.4, 0.002% Tween 20, 0.01% BSA) over 5 mins. A33-loaded tips were tested against three-fold serial dilutions of IgG analytes (27.4 pM-20 nM). The association steps were performed over 10 mins and dissociation steps performed over 20 mins. Buffer control against biosensors loaded with WT-A33 was subtracted from raw data and curves were aligned to baseline. K_D_, k_on_, and k_off_ were determined by global fitting of association and dissociation steps for all dilutions assuming a 1:1 binding model.

### Ectromelia infection and protection studies

Four- to six-week-old female C57BL/6 mice were obtained from Harlan Laboratories (Indianapolis, IN), housed in filter-top microisolator cages, and fed commercial mouse chow and water ad libitum. The mice were housed in an animal biosafety level 3 containment area. The day before challenge, mice were treated i.p. with 100 μg of the specified antibody. Immediately before challenge, mice were anesthetized with 0.1 ml/10 g body weight of ketamine HCl (6 mg/ml) and xylazine (0.5 mg/ml) by i.p. injections. One thousand PFU of ECTV (Moscow strain) in PBS without Ca^2+^ and Mg^2+^ was slowly loaded into nares (5 μl/nare). Mice subsequently were left in situ for 2 to 3 min before being returned to their cages. Mice were monitored daily for mortality and morbidity, as measured by weight change.

### Ethics statement

Animal husbandry and experimental procedures were approved by the Institutional Animal Care and Use Committee of Saint Louis University (ectromelia challenge, protocol number 2082) and by the Department of Laboratory Animal Care and the Animal Care Committee of the La Jolla Institute (VACV challenge, protocol number AP088-BP2-1112). We follow PHS Policy on humane care and use of laboratory animals.

### Accession numbers

The coordinates and structure factors of the A33:Fab complexes have been deposited in the Protein Data Bank (www.rcsb.org) with codes 4LQF, 4LU5, and 4M1G. The nucleotide sequences of the anti-A33 antibodies have been deposited in GenBank, and accession numbers are listed in S5 Table.

## Supporting Information

S1 Table Anti-A33 VACV antibody sequences. (DOCX)

S2 Table Binding kinetics of A33 variants to MAbs A2C7, A20G2, and A27D7. (XLSX)

S3 Table A33 antibody contact details. (DOCX)

S4 Table A33 antibody contact surfaces and shape complementarity. (DOCX)

S5 Table Accession numbers of murine anti-A33 VACV antibody sequences. (DOCX)

S1 Fig Linear peptide ELISA of purified anti-A33 MAbs. Purified anti-A33 MAbs (A2C7, A26C7, A17D7, A25D11, A27D7, A25F2, and A20G2), or no primary antibody (-1’) were tested for binding to overlapping A33 biotinylated 20mer peptides. All seven A33 anti-A33 MAbs bind recombinant A33 protein (A33) but failed to bind any linear peptide, indicating the conformational nature of A33 epitopes.
(TIF)

S2 Fig Comparison of A27D7/A33 CDR contacts with and without bridging waters. Legend is the same as for Fig 5.
(TIF)

S3 Fig Point mutation kinetics analysis by BioLayer Interferometry. Real-time binding curves of MAbs A2C7, A20G2 and A27D7 to immobilized wild-type A33 and indicated A33 mutants are shown. Association (10 min) and dissociation (20 min) steps are represented. Curves are colored according to their specific antigen concentration (bottom right, 20, 10, 5, 2.5, 1.25 nM and 625, 312.5, and 156.2 pM). Association rate (k_on_), dissociation rate (k_off_), affinity constant (K_D_), and fit quality scores are reported in S2 Table. K123 was required for crystal optimization and is far removed from any of the epitope reported herein [40]. Therefore K123A should not influence binding kinetics of any of the MAbs and was chosen as a WT surrogate.
(TIF)

S4 Fig A33 Orthopoxvirus variation profile. (A) Phylogenic cladogram of A33 orthologs. A33 sequence is overall highly conserved. All 106 sequences of A33 orthologs were downloaded from the Poxvirus Bioinformatics Resource Center server (poxvirus.org). Sequence alignment was performed with MEGA5 software using ClustalW with the GONNET similarity matrix and manual refinement. Phylogenic analysis was performed with a global gap removal to exclude gaps from alignments. Maximum Likelihood trees were constructed using the Jones-Taylor-Thornton amino acid substitution model with rates among sites Gamma distributed with Invariant sites. The robustness of trees was evaluated by bootstrap analysis with 1,000 rounds of replication. Non-redundant sequences of the clade framed in red are representative of A33 variation profile for the closest A33^VACV^ orthologs (*Orthopox viridae*; red frame). VACV: vaccinia virus; VARV: variola virus; CMLV: camelpox virus; MPXV: monkeypox virus; MYXV: myxoma virus; CPXV: cowpox virus; ECTV: ectromelia virus; RFV: rabbit fibroma virus; TATV: taterapox virus; LSDV: lumpy skin disease virus; GTPV: goatpox virus; SPPV: sheeppox virus; SWPV: swinepox virus; DPV: mule deer pox virus; YMTV: Yaba monkey tumor virus; TANV: tanapox virus; YLDV: Yaba-like disease virus; MOCV: *Molluscum contagiosum*; ORFV: Orf virus; BSPV: bovine papular stomatitis virus. (B) GENIO/logo representation of the alignment performed with the closest A33^VACV^ orthologs. (C) The model of A33^VACV^ as extracted from A20G2-Fab/A33 complex structure is represented as a blue surface for conserved residues and in red and green for residues that vary among the orthopox species, as defined in (A). As expected, all variable residues are located on the surface. There are a total of 37 potential variations over the whole A33 sequence, among which eighteen can be visualized on the A33 model (residues 98–185). Amino acid positions in C can differ with alignment in B by up to 3, due to different lengths of the sequences.
(TIF)
