# Supplementary material for: Structural and Functional Characterization of Anti-A33 Antibodies Reveal a Potent Cross-Species Orthopoxviruses Neutralizer
Source: PLoS Pathog. 2015 Sep 1;11(9):e1005148. doi: 10.1371/journal.ppat.1005148 (PMC4556652; doi:10.1371/journal.ppat.1005148)
Supplement: S1 Table — (DOCX) [file ppat.1005148.s001.docx]

| **Heavy Chain** | **Sequence ID** | **V-GENE**  **and allele** | **J-GENE and allele** | **CDR1-IMGT** | **CDR2-IMGT** | **CDR3-IMGT** | **J-REGION** |
| --- | --- | --- | --- | --- | --- | --- | --- |
| **1** | A25D11 | IGHV5-12*02 | IGHJ4*01 | GFTLSDYY | ISNGGYKT | ARGMDY | MDYWGQGTSVTVSS |
|  | A20G2 | IGHV5-12*02 | IGHJ4*01 | GFTFSDYY | ISNGGYKT | ARGMDY | MDYWGQGTSVTVSS |
|  |  |  |  |  |  |  |  |
| **2** | A25F2 | IGHV1-54*01 or IGHV1-54*03 or IGHV1S52*01 | IGHJ3*02 | YGFANFL | TNPGSGGA | SRGYDL | WGQGTLVTVSA |
|  |  |  |  |  |  |  |  |
| **3** | A2C7 | IGHV5-12*02 | IGHJ4*01 | GFTFSDYY | ISNSGGNT | ARQWGGAMDY | AMDYWGQGTSVTVSS |
|  |  |  |  |  |  |  |  |
| **4** | A17D7 | IGHV5-17*02 | IGHJ3*01 | GFTFSSFG | ISSGSNTI | ASTGTSYYRPPWFAY | WFAYWGQGTLVTVSA |
|  |  |  |  |  |  |  |  |
| **5** | A27D7 | IGHV2-6-5*01 | IGHJ2*01 | GFSLTDYG | TWGGGTT | AKHKASYNGLDY | DYWGQGTTLTVSS |
|  | A26C7 | IGHV2-6-5*01 | IGHJ2*01 | GFSLTDYG | TWGGGTT | AKHKASYNGLDY | DYWGQGTTLTVSS |
|  |  |  |  |  |  |  |  |
| **Light Chain** | **Sequence ID** | **V-GENE**  **and allele** | **J-GENE and allele** | **CDR1-IMGT** | **CDR2-IMGT** | **CDR3-IMGT** | **J-REGION** |
| **1** | A25D11 | IGKV1-133*01 | IGKJ2*01 | QSLLYSNGKTF | LVS | VQGTHFPYT | YTFGGGTKLEIK |
|  | A20G2 | IGKV1-133*01 | IGKJ2*01 | QSLLYSNGKTY | LVS | VQGTHFPYT | YTFGGGTKLEIK |
|  |  |  |  |  |  |  |  |
| **2** | A25F2 | IGKV1-110*01 | IGKJ1*01 | QSLVHSNGNTY | KVS | SQGTHVPWT | WTFGGGTKLEIK |
|  | A2C7 | IGKV1-110*01 | IGKJ1*01 | QSLIHTNGNTY | KVS | SQSTHIPPWT | WTFGGGTKLEIK |
|  |  |  |  |  |  |  |  |
| **3** | A17D7 | NA | NA | NA | NA | NA | NA |
|  |  |  |  |  |  |  |  |
| **4** | A27D7 | IGKV4-91*01 | IGKJ4*01 | SSISSNY | RTS | QQGSSIPFT | FGSGTKLEIK |
|  | A26C7 | IGKV4-91*01 | IGKJ4*01 | SSINSNY | RTS | QQGSSIPFT | FGSGTKLEIK |
|  |  |  |  |  |  |  |  |
