# Supplementary material for: Structural and Functional Characterization of Anti-A33 Antibodies Reveal a Potent Cross-Species Orthopoxviruses Neutralizer
Source: PLoS Pathog. 2015 Sep 1;11(9):e1005148. doi: 10.1371/journal.ppat.1005148 (PMC4556652; doi:10.1371/journal.ppat.1005148)
Supplement: S3 Table — (DOCX) [file ppat.1005148.s003.docx]

| **MAb A2C7** | | | | | | | | | | | | | |  |  |
| --- | --- | --- | --- | --- | --- | --- | --- | --- | --- | --- | --- | --- | --- | --- | --- |
| **CDR** | **CDR residues** | | **A33 residues** | | | | **H-bonds** | **Salt bridges** | | **VdW** | | **H_2_O** | |  |  |
| **H1** | Y33, Y35 | | Q173, V175, E174 | | | | 2 | 0 | | 17 | | | 0 |  |  |
| **H2** | Y50, N56, Y58 | | Y116, Q173, M118, V175 | | | | 2 | 0 | | 15 | | | 0 |  |  |
| **H3** | Q95, G97, G98 | | D170, Q173 | | | | 0 | 0 | | 15 | | | 0 |  |  |
| **L1** | T27E, N28, Y32, H27D | | D155, D170, S172, F119, G156, N157 | | | | 4 | 0 | | 27 | | | 0 |  |  |
| **L2** | K50 | | D170 | | | | 0 | 1 | | 6 | | | 0 |  |  |
| **L3** | S91, W96, I94 | | Q173, M118 | | | | 3 | 0 | | 20 | | | 0 |  |  |
| **MAb A20G2** | |  | |  | |  | | |  | |  | | |  |  |
| **H1** | Y32, Y33, Y35 | | D168, D170, Q173, S169, E174 | | | | ^1^H: 1  I: 4 | 0 | | H: 15  I: 17 | | | 0 |  |  |
| **H2** | Y50, N52a,  Y55, S52, K56 | | Y116, Q173, V175, Q117, M118, E174 | | | | H: 4  I: 4 | 0 | | H: 45  I: 49 | | | 0 |  |  |
| **H3** | R94 | | D168 | | | | 0 | H: 1  I: 2 | | 0 | | | 0 |  |  |
| **L1** | Y27d, S27e,  N28, Y32 | | D155, G156, F119, G154, N157, V171, S172 | | | | M: 3  L: 5 | 0 | | M: 31  L: 31 | | | 0 |  |  |
| **L2** | none | | none | | | | 0 | 0 | | 0 | | | 0 |  |  |
| **L3** | S91, W96, I94 | | Q173, M118 | | | | M: 3  L: 3 | 0 | | M: 22  L: 21 | | | 0 |  |  |
| **MAb A27D7** | | | | | | | | | | | | | |  |  |
|  |  | | **Chain A** | | **Chain B** | |  |  | |  | | |  |  |  |
| **H1** | G26, F27, S28, D31, Y32, | | E149, T160,  K161, T162,  T163 | | none | | 1 | 1 | | 18 | | | 4 |  |  |
| **H2** | W52, G53, G54, G55, T56, Y58, | | none | | D115, Y116,  Q117, D118,  D121, V175 | | 0 | 0 | | 42 | | | 1 |  |  |
| **H3** | K96, A97, S98, Y99, N100, D101 | | E149, D150, D168, R176 | | D155, Y116, K177 | | 0 | 0 | | 20 | | | 8 |  |  |
| **L1** | Y32 + S29, N31 | | none | | Y116, D150, R176 | | 0 | 0 | | 8 | | | 3 |  |  |
| **L2** | R50, S52, N53, L54, A55, S56, G57, V58, A60 | | Y116, D168,  S169, D170,  V171, S172, Q173,  V175 | | none | | 0 | 0 | | 30 | | | 7 |  |  |
| **L3** | S92, S93, L94 | | none | | Q173, E174,  V175, R176,  M118 | | 0 | 0 | | 28 | | | 0 |  |  |

^1^H, I and L, M refer to the two different heavy and light chains of the ASU.
