# Supplementary material for: Structural and Functional Characterization of Anti-A33 Antibodies Reveal a Potent Cross-Species Orthopoxviruses Neutralizer
Source: PLoS Pathog. 2015 Sep 1;11(9):e1005148. doi: 10.1371/journal.ppat.1005148 (PMC4556652; doi:10.1371/journal.ppat.1005148)
Supplement: S4 Table — (DOCX) [file ppat.1005148.s004.docx]

| **MAb**  **Chain** | **BSA**  **A33 chain A**  **(Å^2^)** | **BSA**  **A33 chain B**  **(Å^2^)** | **BSA**  **A33 dimer**  **(Å^2^)** | **Sc** | **Total BSA**  **(Å^2^)** |
| --- | --- | --- | --- | --- | --- |
| **A2C7** | | | | | |
| LC | 365.1  4.4% | none | none | 0.68 | 699.2  4.9% |
| HC | 334.1  4.1% | none | none | 0.67 |  |
| **A20G2** | | | | | |
| LC | 428.7  4.9% | 401.7  4.7% | none | 0.68 | 802.6/864.3  5.4/5.8% |
| HC | 435.6  5.1% | 400.9  4.8% | none | 0.69 |  |
| **A27D7** | | | | | |
| LC | 259.5  3.1% | 265.8  3.3% | 525.3  5.1% | 0.71 | 1155.5  8.1% |
| HC | 300.4  3.6% | 329.8  4.0% | 630.2  6.0% | 0.74 |  |
