# Supplementary figures and images for: Structural and Functional Characterization of Anti-A33 Antibodies Reveal a Potent Cross-Species Orthopoxviruses Neutralizer
Source: PLoS Pathog. 2015 Sep 1;11(9):e1005148. doi: 10.1371/journal.ppat.1005148 (PMC4556652; doi:10.1371/journal.ppat.1005148)

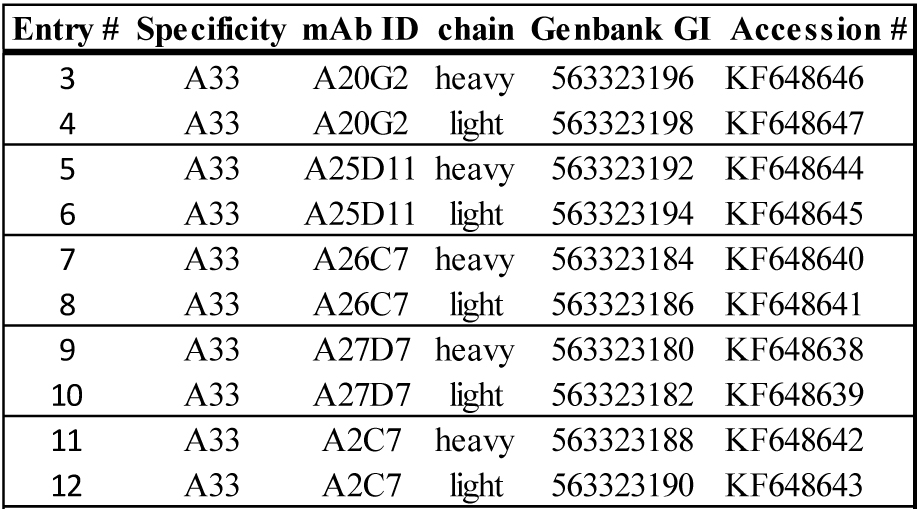

Supplement: S5 Table — (DOCX) [file ppat.1005148.s005.docx]

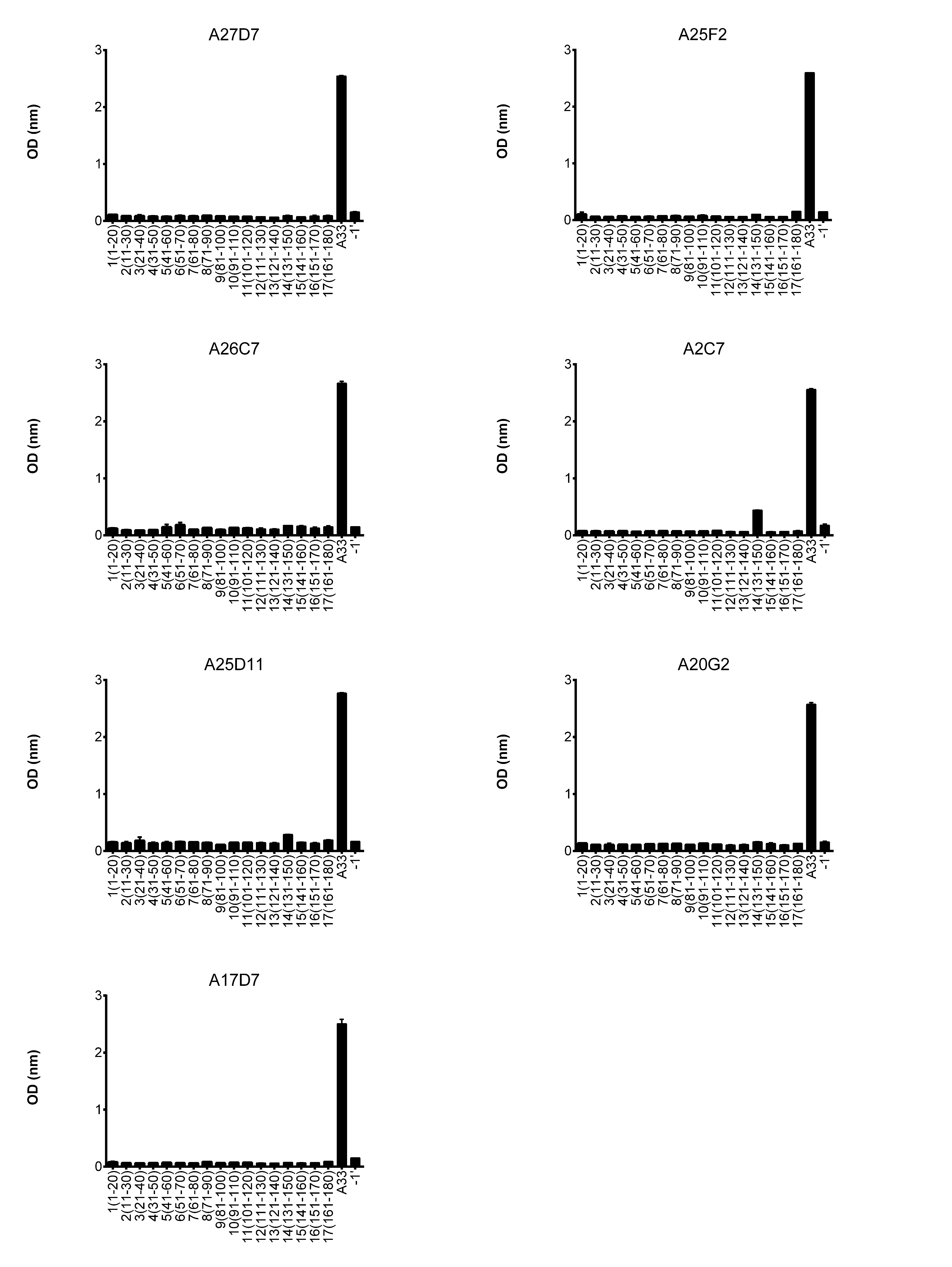

Supplement: S1 Fig — Purified anti-A33 MAbs (A2C7, A26C7, A17D7, A25D11, A27D7, A25F2, and A20G2), or no primary antibody (-1’) were tested for binding to overlapping A33 biotinylated 20mer peptides. All seven A33 anti-A33 MAbs bind recombinant A33 protein (A33) but failed to bind any linear peptide, indicating the conformational nature of A33 epitopes. (TIF) [file ppat.1005148.s006.tif]

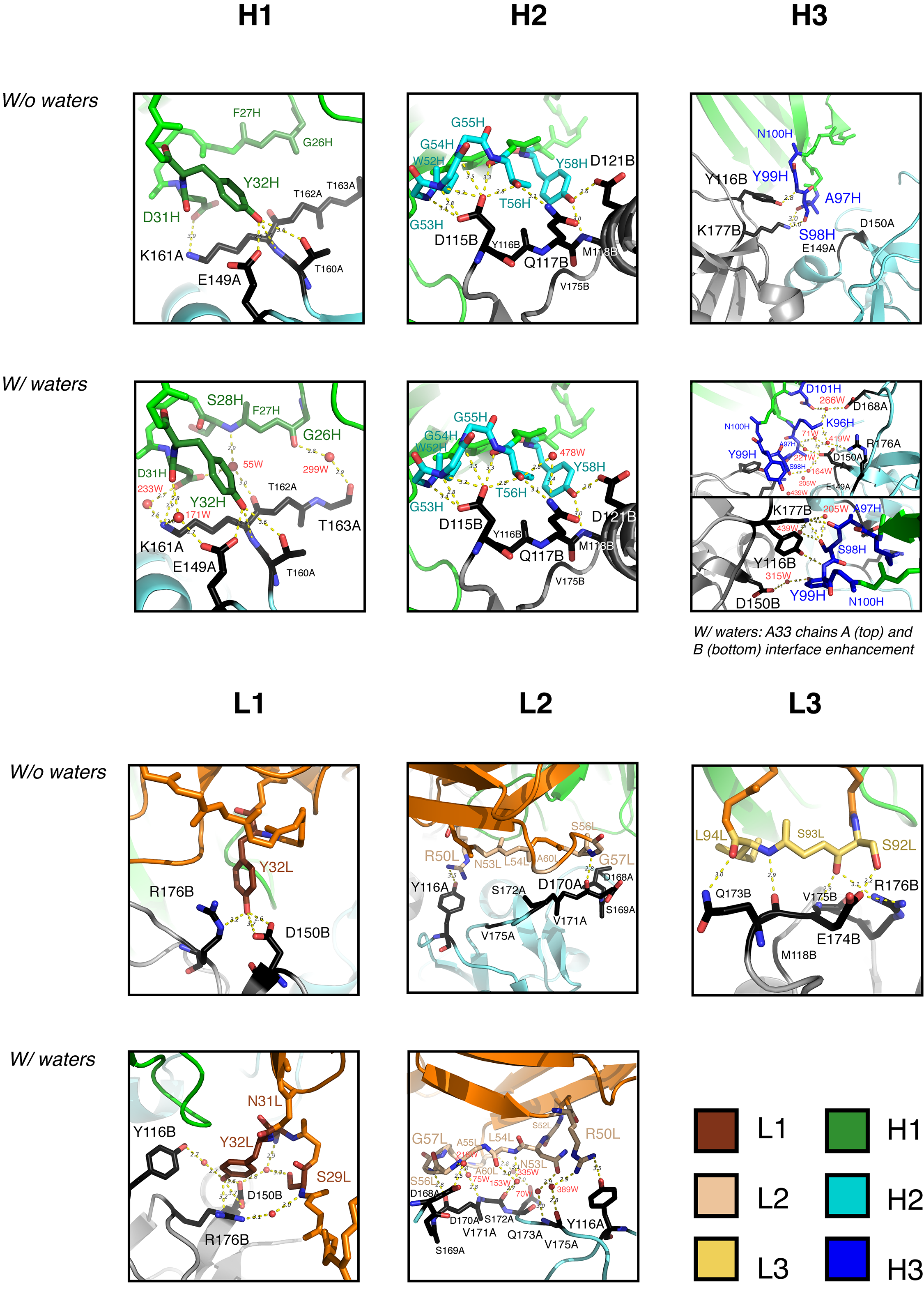

Supplement: S2 Fig — Legend is the same as for Fig 5. (TIF) [file ppat.1005148.s007.tif]

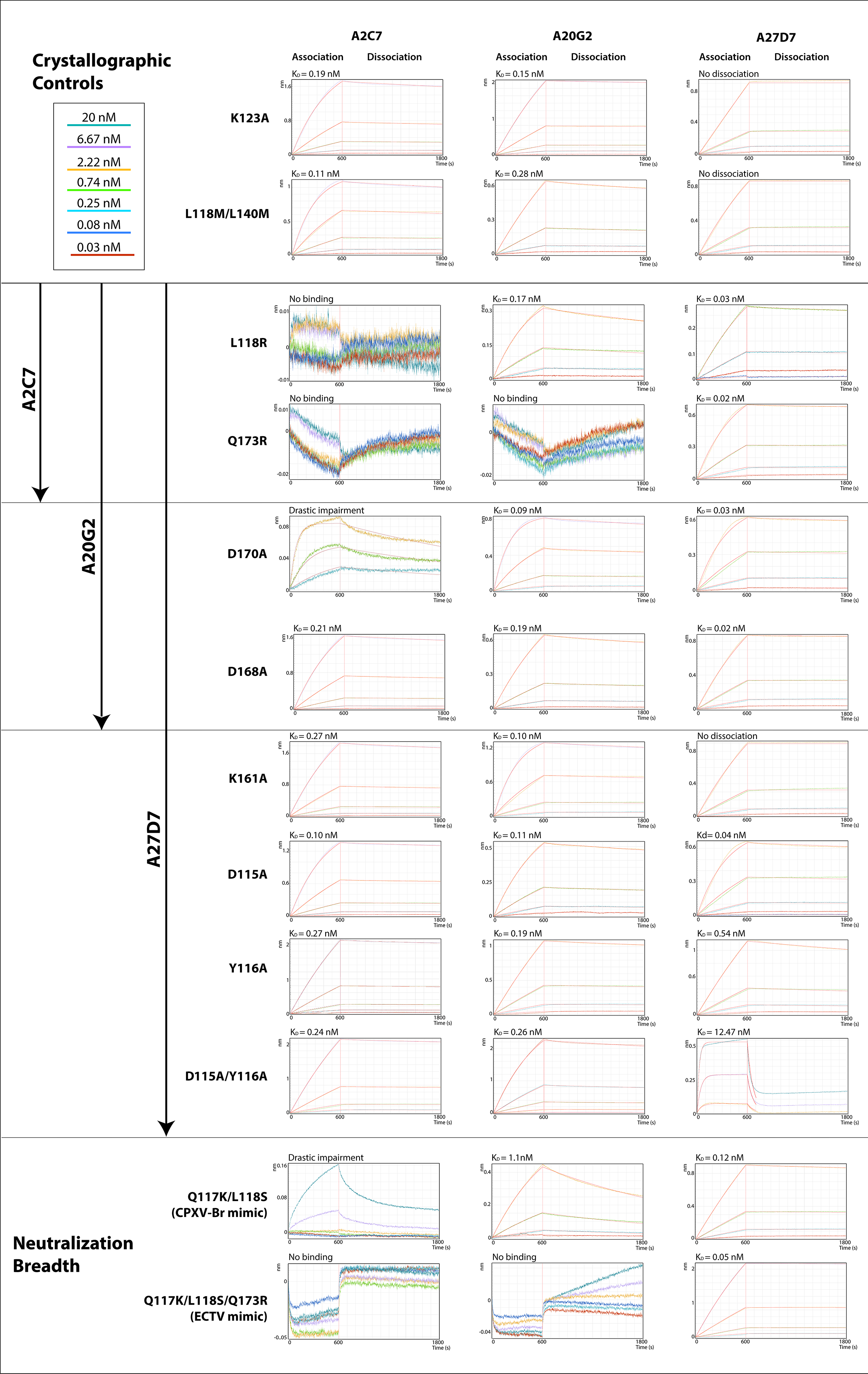

Supplement: S3 Fig — Real-time binding curves of MAbs A2C7, A20G2 and A27D7 to immobilized wild-type A33 and indicated A33 mutants are shown. Association (10 min) and dissociation (20 min) steps are represented. Curves are colored according to their specific antigen concentration (bottom right, 20, 10, 5, 2.5, 1.25 nM and 625, 312.5, and 156.2 pM). Association rate (kon), dissociation rate (koff), affinity constant (KD), and fit quality scores are reported in S2 Table. K123 was required for crystal optimization and is far removed from any of the epitope reported herein [40]. Therefore K123A should not influence binding kinetics of any of the MAbs and was chosen as a WT surrogate. (TIF) [file ppat.1005148.s008.tif]

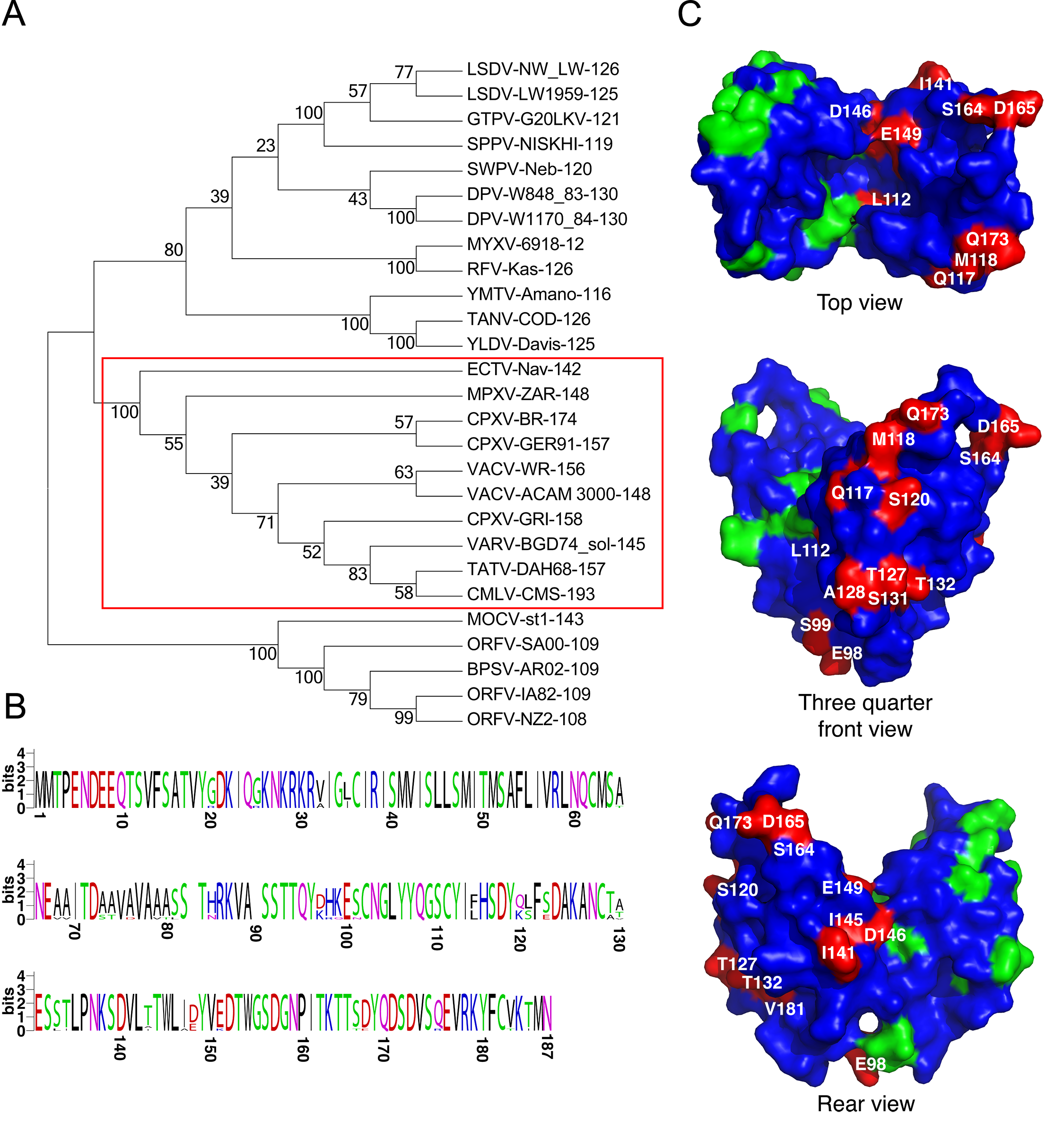

Supplement: S4 Fig — (A) Phylogenic cladogram of A33 orthologs. A33 sequence is overall highly conserved. All 106 sequences of A33 orthologs were downloaded from the Poxvirus Bioinformatics Resource Center server (poxvirus.org). Sequence alignment was performed with MEGA5 software using ClustalW with the GONNET similarity matrix and manual refinement. Phylogenic analysis was performed with a global gap removal to exclude gaps from alignments. Maximum Likelihood trees were constructed using the Jones-Taylor-Thornton amino acid substitution model with rates among sites Gamma distributed with Invariant sites. The robustness of trees was evaluated by bootstrap analysis with 1,000 rounds of replication. Non-redundant sequences of the clade framed in red are representative of A33 variation profile for the closest A33VACV orthologs (Orthopox viridae; red frame). VACV: vaccinia virus; VARV: variola virus; CMLV: camelpox virus; MPXV: monkeypox virus; MYXV: myxoma virus; CPXV: cowpox virus; ECTV: ectromelia virus; RFV: rabbit fibroma virus; TATV: taterapox virus; LSDV: lumpy skin disease virus; GTPV: goatpox virus; SPPV: sheeppox virus; SWPV: swinepox virus; DPV: mule deer pox virus; YMTV: Yaba monkey tumor virus; TANV: tanapox virus; YLDV: Yaba-like disease virus; MOCV: Molluscum contagiosum; ORFV: Orf virus; BSPV: bovine papular stomatitis virus. (B) GENIO/logo representation of the alignment performed with the closest A33VACV orthologs. (C) The model of A33VACV as extracted from A20G2-Fab/A33 complex structure is represented as a blue surface for conserved residues and in red and green for residues that vary among the orthopox species, as defined in (A). As expected, all variable residues are located on the surface. There are a total of 37 potential variations over the whole A33 sequence, among which eighteen can be visualized on the A33 model (residues 98–185). Amino acid positions in C can differ with alignment in B by up to 3, due to different lengths of the sequences. (TIF) [file ppat.1005148.s009.tif]
